# Design, Synthesis and Biological Evaluation of 2-(2-Amino-5(6)-nitro-1*H*-benzimidazol-1-yl)-*N*-arylacetamides as Antiprotozoal Agents

**DOI:** 10.3390/molecules22040579

**Published:** 2017-04-04

**Authors:** Emanuel Hernández-Núñez, Hugo Tlahuext, Rosa Moo-Puc, Diego Moreno, María Ortencia González-Díaz, Gabriel Navarrete Vázquez

**Affiliations:** 1 CONACYT, Departamento de Recursos del Mar, Centro de Investigación y de Estudios Avanzados del IPN, Unidad Mérida, Mérida 97310, Yucatán, Mexico; 2 Centro de Investigaciones Químicas, Universidad Autónoma del Estado de Morelos, Cuernavaca 62210, Morelos, Mexico; tlahuext@uaem.mx; 3 Unidad Interinstitucional de Investigación Médica, Instituto Mexicano del Seguro Social/Universidad Autónoma de Yucatán, Mérida 97150, Yucatán, Mexico; moopuc@gmail.com; 4 Facultad de Ingeniería, Universidad Autónoma de Yucatán, Mérida 97310, Yucatán, Mexico; diegomorenor@gmail.com; 5 CONACYT, Centro de Investigación Científica de Yucatán, Mérida 97200, Yucatán, Mexico; maria.gonzalez@cicy.mx; 6 Facultad de Farmacia, Universidad Autónoma del Estado de Morelos, Cuernavaca 62209, Morelos, Mexico; gabriel_navarrete@uaem.mx

**Keywords:** benzimidazole, antiprotozoal, NMR prediction

## Abstract

Parasitic diseases are a public health problem affecting millions of people worldwide. One of the scaffolds used in several drugs for the treatment of parasitic diseases is the benzimidazole moiety, a heterocyclic aromatic compound. This compound is a crucial pharmacophore group and is considered a privileged structure in medicinal chemistry. In this study, the benzimidazole core served as a model for the synthesis of a series of 2-(2-amino-5(6)-nitro-1*H*-benzimidazol-1-yl)-*N*-arylacetamides **1**–**8** as benznidazole analogues. The in silico pharmacological results calculated with PASS platform exhibited chemical structures highly similar to known antiprotozoal drugs. Compounds **1**–**8** when evaluated in silico for acute toxicity by oral dosing, were less toxic than benznidazole. The synthesis of compounds **1**–**8** were carried out through reaction of 5(6)-nitro-1*H*-benzimidazol-2-amine (**12**) with 2-chlroactemides **10a**–**h**, in the presence of K_2_CO_3_ and acetonitrile as solvent, showing an inseparable mixture of two regioisomers with the -NO_2_ group in position 5 or 6 with chemical yields of 60 to 94%. The prediction of the NMR spectra of molecule **1** coincided with the experimental chemical displacements of the regioisomers. Comparisons between the NMR prediction and the experimental data revealed that the regioisomer *endo*-1,6-NO_2_ predominated in the reaction. The in vitro antiparasitic activity of these compounds on intestinal unicellular parasites (*Giardia intestinalis* and *Entamoeba histolytica*) and a urogenital tract parasite (*Trichomonas vaginalis*) were tested. Compound **7** showed an IC_50_ of 3.95 μM and was 7 time more active against *G. intestinalis* than benznidazole. Compounds **7** and **8** showed 4 times more activity against *T. vaginalis* compared with benznidazole.

## 1. Introduction

Parasitic diseases are a public health problem affecting millions of people worldwide [1]. Parasites are eukaryotic and share common features with their mammalian host. This has motivated the search for effective and selective drugs, although the task remains a difficult one. While significant efforts have been made in the discovery of new therapeutic targets that are more selective and more potent, these can also come with side-effects that can be severe [2]. The current status of anti-parasitic drug research that can be classified in drug repositioning (old drugs for pharmacological therapies to current diseases) [3], the search of new drugs obtained of the nature (plants, organism’s marine, terrestrial, bacteria, to mention some) [4], the rational design of drugs (medicinal chemistry) [5] and the evaluation of new chemical entities in in silico evaluations [6]. Benzimidazole, a heterocyclic aromatic chemical compound, is a crucial pharmacophore group and a privileged structure in medicinal chemistry. Many heterocyclic nuclei are present in antiparasitic drugs, such as 5(6)-substituted benzimidazoles and 2-aminonitrobenzimidazoles [7,8]. The 2-aminobenzimidazole core is found in albendazole, the most common antiparasitic drug and the drug of choice for anti-infectious chemotherapy against anaerobic protozoa and other parasites [9]. Benznidazole [Bnz, Rosanil], a 2-nitroimidazole derivative, is an important drug for Chagas disease, and has been used in other parasitic diseases [10]; For example: Nava-Zuazo et al. (2014) proposed the use of Bnz as an alternative treatment against *Giardia intestinalis*, *Entamoeba histolytica* and *Trichomonas vaginalis* in in vitro experiments [11] and Bernandino et al. used the drug against four *Leishmania* species [12]. Currently, the pharmaceutical industry uses old drugs with known pharmacokinetic parameters for new diseases in order to save time in the drug development, a process called drug repositioning [13]. Bnz’s mode of action is related to reductive metabolism. It functions as a prodrug and must be activated by an NADH-dependent, mitochondrially localized, bacterial-like, type I nitroreductase [14]. Several compounds containing the 5(6)-nitro or 2-amino-benzimidazole scaffold have been used as antiparasitic [15], antimycobacterial [16], antimicrobial [17] and antifungal [18], anthelmintic [19], antitumoral [20], antioxidant [21], analgesic and anti-inflammatory[22], antihypertensive and vasorelaxant [23] agents. As a part of our search for basic information about the structural requirements for new antiparasitic activities of an old drug (Bnz), we synthesized a series of novel 2-(2-amino-5(6)-nitro-1*H*-benzimidazol-1-yl)-*N*-arylacetamide benzoanalogues of this drug. The in vitro antiparasitic activity of these compounds on intestinal unicellular parasites (*Giardia intestinalis* and *Entamoeba histolytica*) and a urogenital tract parasite (*Trichomonas vaginalis*) are reported in this paper.

## 2. Results and Discussion

### 2.1. Drug Design of Benznidazole Derivatives

The compounds shown in Table 1 were designed based on the structures of benznidazole, a drug used to treat trypanosomiasis, and widely recommended as an antiprotozoal agent [24,25].

**Scheme 1:**
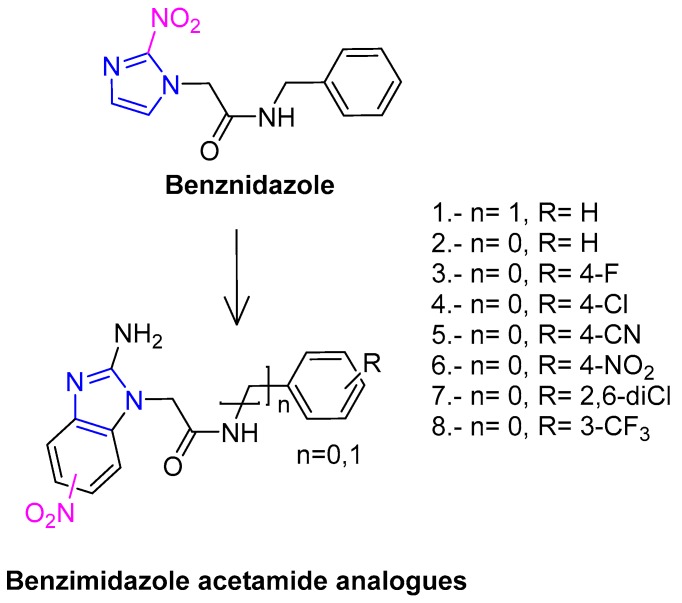
Drug design of benznidazole analogues.

Common constituents of these compounds are the imidazole ring and the presence of the nitro group, which is located in 2-position. The first design consideration was to join the benznidazole to generate a series of imidazole derivatives that were reported in previous studies [26]. The second consideration in this work was the introduction of an aromatic ring between the nitro group and the imidazole following the principle of vinylogy [27] to generate 5(6)-nitrobenzimidazole derivatives. Finally, the third consideration was to change the nitro group present in 2-position for the amine group, favoring the formation of hydrogen bonds and increasing solubility in aqueous systems [28] (Scheme 1).

### 2.2. Evaluation in Silico (PASS and ACDTox/Suite)

Predictive values concerning antiparasitic activities were obtained by comparing the chemical structures of the compounds designed with the structures or substructures of more than 30,000 well-known biologically active drugs. Prediction results are presented as estimates of the probability, *Pa*, that the compounds are active and *Pi*, the probability that compounds are not active. For *Pa* values = 0.7, the corresponding compound is very likely to reveal this activity in experiments, however the chance of the compound being the analogue of a known pharmaceutical agent is also high. For *Pa* values between 0.5 and 0.7, the compound is likely to reveal this activity in experiments and the compound exhibits less similarity to the known pharmaceutical agents. For *Pa* values lower than 0.5, the compound is unlikely to reveal this activity in experiments, but if the presence of this activity is confirmed in experiments, the compound might be a new biologically active chemical entity. Predictive values for 2-(2-amino-5-nitro-1*H*-benzimidazol-1-yl)-*N*-arylacetamide **1**–**8** are summarized in Table 1. These show biological activities against trichomonas. *Pa* values estimated for trichomonas activity were lower than 0.5 for all compounds. For benznidazole the *Pa* values estimated for trichomonas activity were 0.497 and *Pa* values estimated for antiprotozoal (*Trichomonas vaginalis*) activity were 0.705. These results indicated that 2-(2-amino-5-nitro-1*H*-benzimidazol-1-yl)-*N*-arylacetamide **1**–**8** exhibited chemical structures highly similar to known antiprotozoal drugs.

Computational prediction of toxicity [29] has been performed in drug development in order to anticipate potentially harmful substances. The toxicity parameters of all the benznidazole compounds were calculated with ACD/Tox Suite software (v. 2.95) [30].

The acute toxicity of a chemical is defined as a dose that is lethal to 50% of treated animals (LD_50_). The acute toxicity can be viewed as a ‘cumulative potential’ to cause various acute effects and death of animals [31]. In these predictions, all compounds evaluated in silico in mice by oral dosing were less toxic than benznidazole. However, intraperitoneal (i.p.) route of administration the compounds are shown to be more toxic than benznidazole.

### 2.3. Chemical Synthesis and Characterization

**Scheme 2:**
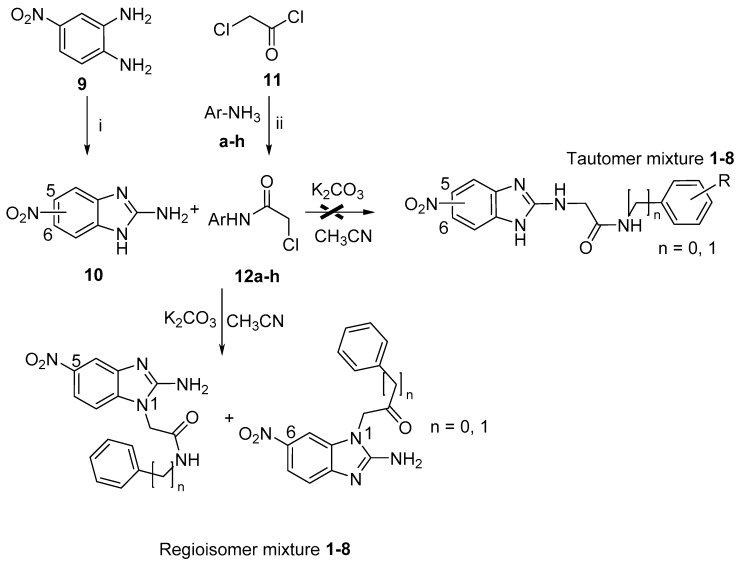
Reagents: (**i**) BrCN, Diglime/water (4:1); (**ii**) NaHCO_3_, acetone.

The appropriately substituted α-chloroacetamides **12a**–**h**, were synthesized by condensation of arylanilines **a**–**h** with 2-chloroacetyl chloride (**11**) (Scheme 2). In the methodology, we used methylene chloride as the solvent and triethylamine as the base, producing the desired compounds with yields ranging from 70%–90%. The reaction conditions and product yields are listed in a previous article [26]. Compound **10** was prepared as shown in Scheme 1 [32]. Treatment of 4-nitro-1,2-phenylenediamine (**9**) with cyanogen bromide in a mixture of diglyme/water (4:1) at 90 °C gave 5(6)-nitro-1*H*-benzimidazol-2-amine (**10**). The synthesis of compounds **1**–**8** were carried out through the reaction of 5(6)-nitro-1*H*-benzimidazol-2-amine (**10**) with 2-chloroacetamides **12a**–**h**, in the presence of K_2_CO_3_ and acetonitrile as solvents, as shown in Scheme 2. Solid compounds were purified by recrystallization or by column chromatography and the structure of the pure compounds was established by spectroscopic data. The compounds **1**–**8** were obtained in good yields (Table 2).

The chemical structures of the synthesized compounds were confirmed based on their spectral data (NMR and mass spectra), and their purity ascertained by microanalysis. The elemental analysis was 5 ppm less than the theoretical values obtained for FAB-MS. Physical constants of the title compounds are shown in Table 2.

**Figure 1:**
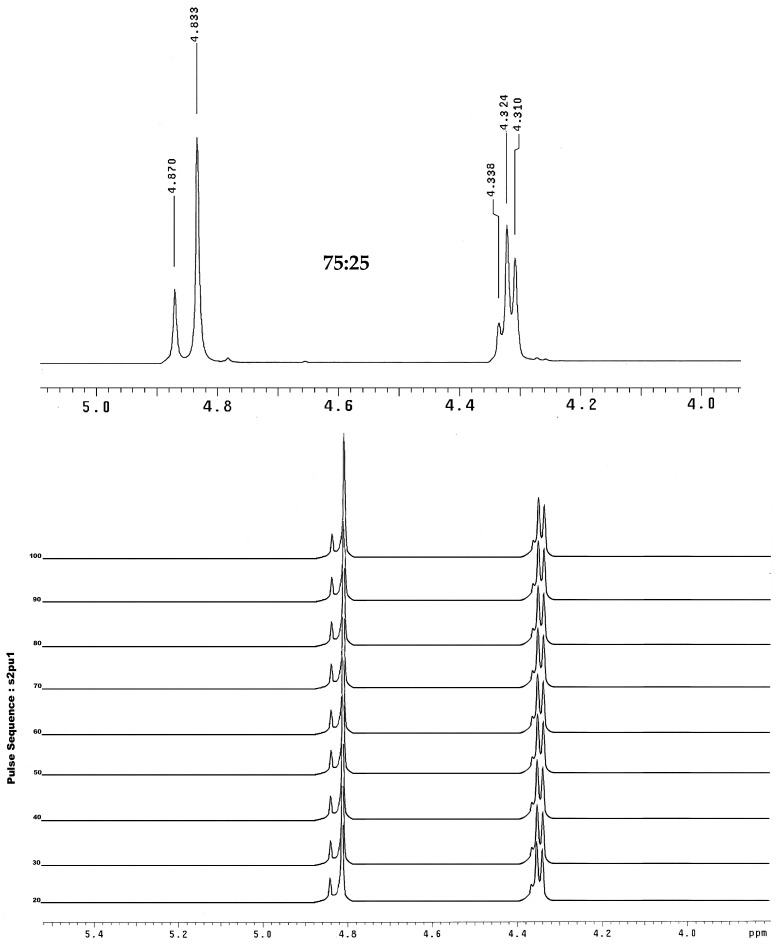
(**a**) ^1^H-NMR spectrum for compound **1** in the region between 4 and 5 ppm and (**b**) ^1^H-NMR spectra acquired at different temperature from 20 to 100 °C (400 MHz, DMSO-*d*_6_).

In the nuclear magnetic resonance spectra, two possible products were observed by ^1^H- and ^13^C-NMR. Figure 1a shows the ^1^H-NMR spectrum of compound **1**, where the signals corresponding to the benzylic protons found at approximately 4.4 ppm and the methylene hydrogens *alpha* to the carbonyl group at 4.8 ppm are observed in a ratio of 75:25.

Based on the NMR data, to confirm the formation of regioisomers a variable temperature NMR experiment was performed (Figure 1b), in which the existence of a tautomer mixture would be confirmed by rapid interconversion between the two components of the mixture. The coalescence temperature would be where a single signal is observed for each of the protons of the structure. In this experiment, coalescence with increasing temperature was not observed, leading to the conclusion that the mixture analyzed did not correspond to the formation of tautomers, but rather to the generation of regioisomers (Scheme 1).

**Figure 2:**
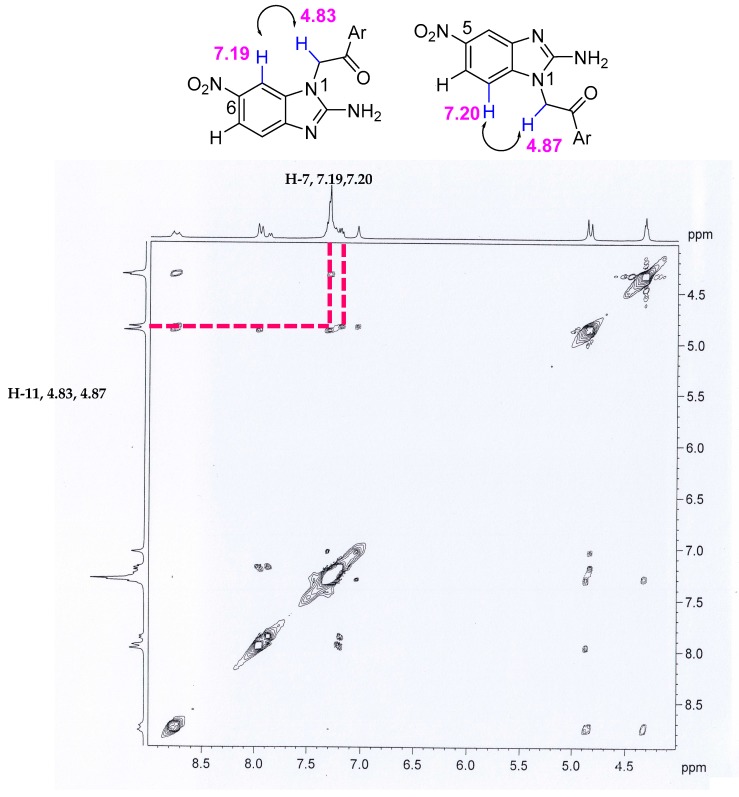
NOESY NMR experiment (mixing time 100 ms, temperature 25 °C, Varian Oxford 400 MHz).

Thus, the existence of a mixture of regioisomers was proposed and derivative allocation was performed. The formation of the regioisomer mixture could be explained by the possible formation of hydrogen bonds between the carbonyl oxygen group and the hydrogen of the amino group. This favors the formation of both regioisomers and the hydrogen bond, which gives them a conformation in three dimensions composed of a 6-membered ring, anchoring the conformations observed for these compounds and reducing the energy to a global minimum to stabilize the molecules (Figure 2). To strengthen the hypothesis of the formation of a mixture of regioisomers, two-dimensional NMR experiments were performed, in which a correlation of protons was observed in the NOESY spectrum at 4.8 ppm (–CH_2_) with the corresponding aromatic proton of the benzimidazole, in this case with the H-7, indicating that the possible structure is binding to the N^1^, which would generate a mixture of regioisomers (Figure 2).

In the structural elucidation of the benzimidazole derivatives, the signals (^1^H-NMR; δ ppm) of the respective protons of the compounds were verified based on their chemical shifts, multiplicities and coupling constants. In compounds **1**–**8**, the aromatic region of the ^1^H-NMR spectrum contained signals ranging from δ 7.04–8.24 ppm, attributable to H-2′, H-3′, H-5′ and H-6′, of the 4-substituted benzene ring. In all compounds, observations included a characteristic pattern for NHCH_2_CO (H-11), a singlet methylene signal ranging from 4.83 to 5.11, and a broad signal ranging from 7.03 to 7.63, attributable to the NH_2_ of the heterocyclic ring. One peak (*J*_m_ = 2.0 Hz) was observed for compounds **1**–**8**, assigned to hydrogen H-4 or H-7 found in aromatic benzimidazole (Table 3).

**Scheme 3:**
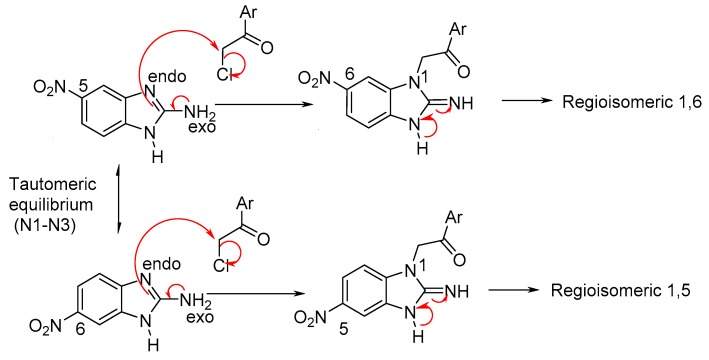
Mechanism of the formation of reigoisomers mixture 1,5 and 1,6.

Scheme 3 describes the proposed reaction mechanism for the formation of the regioisomers. Initially, the electron pair of the nitrogen *exo* located in C-2 is introduced and is directed towards the *N*-3 *endo* (tautomeric electron transfer to Petrova et al.) [33]. The anion formed nucleophilic ally displaces the chlorine from the amide, where the *N*^1^ enters its electron pair system to regenerate aromaticity, leading to the formation of the reigoisomer mixture. These were inseparable from one another using conventional methods, such as crystallization and open column chromatography.

Some examples of mixtures of regioisomers have been previously described in the literature, for instance Chen and Willis (2015) reported that a combination of arynes, generated using fluoride from the corresponding 2-(trimethylsilyl)aryl triflates and 3-hydroxy-4-aminothiadiazoles, leads to the selective formation of 3-amino-substituted benzo[d]isothiazoles to obtain an inseparable mixture of regioisomers [34]. The diprop-2-ynylamines **4a**–**c** were treated with methyl prop-2-ynyl ether under standard conditions. The obtained product, isoindolinone, was isolated by column chromatography as an inseparable 1:1 mixture of regioisomers [35]. On the other hand, in a study of anticancer compounds, an inseparable mixtures of 2 regioisomers were obtained from 6-[*N*-(2-*t*-butyldimethylsilyloxyethyl)-*N*-methylamino]-2-bromo-3-methoxy-1,4,5,8-triptycenetetraone; and 6-[*N*-(4-hydroxybutyl)-*N*-methylamino]-2-bromo-3-methoxy-1,4,5,8-triptycenetetraone by silica gel column chromatography [36]. In addition, Meng et al. reported the synthesis of a regioisomer mixture (1:1 ratio) of 5(6)-methyl-1-(1-phenylethyl)-1*H*-benzo[d]imidazole [37].

### 2.4. NMR Prediction

The ^1^H and ^13^C theoretical and experimental chemical shifts (δ), isotropic shielding values (σ) and the assignments of fourth compounds are included in Table 3. The observed ^1^H and ^13^C-NMR spectra of the compounds **1a** and **1b** in DMSO-*d*_6_ solvent are shown in the Table 3, respectively. All calculations of the NMR shielding constants have been performed using gauge-including atomic orbitals (GIAO) approximation provided by GAUSSIAN 09,[38] in DMSO solvent. Nwchem program package (version 6.5) was employed [39]. Geometry optimizations were carried out at the DFT level with the B3LYP exchange-correlation functional [40] and Def2-TZVP [41] basis set. Harmonic vibrational frequencies were also analyzed at the same level to characterize the nature of the stationary molecules. The isotropic shielding values were used to calculate the isotropic chemical shifts with respect to tetramethylsilane (TMS).

The ^1^H and ^13^C chemical shifts are referenced from the DMSO-*d*_6_ solvent resonances, which are assigned as 2.50 and 39.5 ppm, respectively. The experimental chemical shifts of proton attached to nitrogen for the compounds **1a** and **1b** were shifted towards downfield due to the hydrogen bonding interaction and the signals were observed at 7.03 and 8.73 ppm, respectively. The computed NMR chemical shifts of NH protons of compounds **1a** and **1b** were found at 5.54, 5.82, 5.15 and 5.32 ppm, respectively. The calculated value of N-H proton for compound **1** remains unacceptable apart from the experimental values, since the chemical shift associated with this proton is not correctly described by the continuum model. For the compounds **1a** and **1b**, the difference between the theoretical and experimental N-H chemical shifts is probably caused by intermolecular hydrogen bonding. This discrepancy can be explained by the DFT calculations belonging to an isolated molecule in gas phase while the experimental data belong to the same molecule in solid state phase. The experimental chemical shift of methylene protons (H-11) was shifted towards downfield due to the electronegativity of nitrogen atom and the signals were observed at 4.83 and 4.87 ppm for the compounds **1a** and **1b**, respectively. Corresponding signals for the phenyl substituted methylene protons (H-14) were also observed at the 4.32 and 4.33 4 ppm, respectively. The computed NMR chemical shifts of methylene protons (H-11) of compounds **1a** and **1b** were found at 4.30 and 4.31, 4.61 and 4.62 ppm, respectively. The difference between the chemical shifts was 0.21 with an estimated error of 4.5% for the method used in this calculation for these protons. The protons of the benzimidazole group were experimentally found at 7.93, 7.87 and 7.19 ppm in the DMSO solution for compound **1a** (H-4, H-6 and H-7, respectively); at 7.20, 7.95 and 7.97 ppm for compound **1b** (H-4, H-5 and H-7, respectively). For comparison, our theoretical calculations yielded in 7.52, 8.57 and 8.10 ppm for compounds **1b**, and 6.75, 8.38 and 8.74 ppm for **1a**. Calculated ^1^H-NMR peaks are in agreement with both experimental (Table 3).

For the chemical displacements of ^13^C, the experimental and theoretical displacements were compared, revealing only small differences (Table 3). It was concluded from the data collected in Table 3 that experimental and theoretical values of ^13^C-NMR were more compatible than those values acquired for ^1^H-NMR.

With regard to the protons of the benzimidazole group for the tautomers **1c** and **1d**, the estimated error between the experimental values and theoretical calculations is about 11.1% (see Table 3 for chemical shift values) suggesting that these isomers were not formed. The ^13^C theoretical data do not correspond to those obtained experimentally (Table 3).

### 2.5. In Vitro Antiprotozoal Assays

In this study eight new 2-(2-amino-5-nitro-1*H*-benzimidazol-1-yl)-*N*-Arylacetamides **1**–**8** were tested in vitro as antiprotozoal agents against *G. intestinalis*, *T. vaginalis* and *E. histolytica*. The main features of these compounds are the substitution of the hydrogen atom at position 3, 4 or 6, 7 by different fluorine, nitro, chloro, trifluoromethyl and cyanide substituent groups which were used to determine bioisosteric equivalence, enhancement of solubility, and potential antiprotozoal activity.

The results of the biological assays are shown in Table 2, All synthesized compounds were more active against *G. intestinalis* than benznidazol. Compound **8** is observed to be 7 times more active against this parasite than Bnz due to the presence of the trifluoromethyl group in the *meta* position.

For *T. vaginalis*, benznidazole presents an IC_50_ of 18.62 μM. Conversely, compounds **4** and **7** are three times more potent than benznidazole. We can see that the activity increases when we have an –NO_2_ group (compound **4)** or 2,6-dichloro substituent (compound **7**). These groups are needed to maintain trichomonacidal activity in subsequent molecular changes. For *Entamoeba histolytica*, benznidazole was the most active, presenting an IC_50_ of 4.27 μM, and all synthesized compounds were less active than benznidazole. It is observed that compound **4**, with a second –NO_2_ group as a substituent, obtained the best amoebicidal activity of the series.

The compounds shown in Table 2 are fully compatible with Lipinski’s rule [42,43], which should allow for the development of additional antiprotozoal analogues. Their advantages include:

- Physical properties known to be compatible with desirable pharmacokinetics (low molecular weight, favorable Clog P, favorable hydrogen bond donating and accepting capabilities);
- Potency and efficacy, with IC_50_ values at the low micromolar level;
- Simple synthetic access and thus low production costs;
- Bioisosteric groups improving the likelihood of reasonable solubility.

When performing the in silico evaluation with the PASS@ program where the Activity Probability (Pa) is predicted and the data are sorted from highest to lowest, they maintain the following sequence: **6**(4-NO_2_-Ph) > **2**(Ph) > **1**(Bn) > **4**(4-Cl-Ph) > **7**(2,6-diCl-Ph) > **3**(4-F-Ph) > **5**(4-CN-Ph) > **8**(3-CF_3_-Ph); In contrast to the experimental data for *Trichomonas vaginalis*, the following scheme was found: **8**(3-CF_3_-Ph) > **7**(2,6-diCl-Ph) > **6**(4-NO_2_-Ph) > **5**(4-CN-Ph) > **1**(Bn) > **4**(4-Cl-Ph) > **3**(4-F-Ph) > **2**(Ph), correlation of in silico-in vitro activity is low, indicated by a ratio **7** (2,6-dichlorophenyl) and **5** (4-CN-Ph). Biological activity prediction programs (PASS@) provide information on the relationship of structure and biological activity before developing the synthesis, but it is necessary to perform the experiments to test them.

In summary, we synthesized a series of benzimidazole derivatives obtaining an inseparable regioisomers mixture, with the 1,6-substituted compound as predominant isomer. All compounds show good antiprotozoal profile against *G. intestinalis* and *T. vaginalis* in comparison with benznidazole.

## 4. Materials and Methods

### 4.1. Chemistry

Melting points were determined on an EZ-Melt MPA120 automated melting point apparatus from Stanford Research Systems (Sunnyvale, CA, USA) and are uncorrected. Reactions were monitored by TLC on 0.2 mm precoated silica gel 60 F254 plates (E. Merck KGaA, Darmstadt, Germany). ^1^H-NMR (400 MHz) and ^13^C-NMR (100 MHz) spectra were recorded on Varian Oxford (Varian, Palo Alto, CA, USA). Chemical shifts are given in ppm relative to tetramethylsilane (Me_4_Si, δ = 0) in DMSO-*d*_6_; values are given in Hz. The following abbreviations are used: s, singlet; d, doublet; q, quartet; dd, doublet of doublet; t, triplet; m, multiplet; br, broad signal. MS were recorded on a JEOL JMS-700 spectrometer (JEOL, Tokyo, Japan) by Fast atom bombardment [FAB (+)]. Clog *p* values were obtained using Molinspiration (www.molinspiration.com; Slovensky Grob, Slovak Republic). Predictive values of antiprotozoal activities were also investigated using the PASS chemistry software server (http://www.ibmh.msk.su/PASS/), per the mathematical model and database developed by Lagunin et al. [44]. Starting materials were commercially available from Sigma Aldrich (Sigma-Aldrich, St. Louis, MI, USA) and used without purification.

### 4.2. General Method of Synthesis of 2-(2-Amino-5(6)-nitro-1H-benzimidazol-1-yl)-N-Arylacetamides ***1***–***8***

The appropriate *N*-arylacetamide (1.2 mol) was added dropwise to a solution of 5-nitro-1*H*-benzoimidazol-2-ylamine (1.0 mol) in acetonitrile (30 mL) and potassium carbonate (2.2 mol) at 90 °C. The mixture was stirred at reflux for between 5 and 18 h. The solvent was removed under vacuum and the residue was suspended in water. The precipitates were filtered and dried. Crude compounds were recrystallized from DMSO:H_2_O mixture (50:50) or purified by column chromatography.

*2-(2-Amino-5(6)-nitro-1H-benzimidazol-1-yl)-N-benzylacetamide* (**1**). Yield: 90.1%; m.p. 268–271 °C; Yellow solid, ^1^H-NMR and ^13^C-NMR refer to a 75:25 mixture of regioisomers.

*75% Regioisomer of (2-(2-amino-6-nitro-1H-benzimidazol-1-yl)-N-benzylacetamide* (**1a**). ^1^H-NMR. (400 MHz, DMSO-*d*_6_) δ 4.32 (2H, d, *J_NH-H_* = 5.6 Hz, NHCH_2_Ph); 4.83 (2H, s, NHCH_2_CO); 7.03 (2H, br, NH_2_); 7.19 (1H, d, *J_orto_* = 8.4 Hz, H-7); 7.23–7.36 (5H, m, Ph); 7.87 (1H, dd, *J_meta_* = 2.4 Hz, *J_orto_* = 8.4 Hz, H-6); 7.93 (d, *J_meta_* = 2.0 Hz, 1H, H-4); 8.73 (t, *J_NH-H_* = 6.0 Hz, 1H, CONH) ppm. ^13^C-NMR (100 MHz, DMSO-*d*_6_) δ 42.4 (NHCH_2_Ph); 44.9 (NHCH_2_CO); 107.1 (C-7); 109.3 (C-6); 114.7 (C-4); 126.8 (C-4′); 127.2 (2C, C-2′, C-6′); 128.2 (2C, C-3′, C-5′); 138.8 (C-1′); 140.1 (C-5); 141.9 (C-7a); 142.8 (C-3a); 158.1(C-2); 165.9 (C=O) ppm.

*25% Regioisomer of (2-(2-amino-5-nitro-1H-benzimidazol-1-yl)-N-benzylacetamide* (**1b**). ^1^H-NMR (400 MHz, DMSO-*d*_6_) δ 4.33 (2H, d, *J_NH-H_* =5.6 Hz, NHCH_2_Ph); 4.87 (2H, s, NHCH_2_CO); 7.03 (2H, br, NH_2_); 7.20 (1H, d, *J_orto_* = 8.4 Hz, H-4); 7.23–7.36 (m, 5H, Ph); 7.95 (1H, dd, *J_meta_* = 2.6 Hz, *J_orto_* = 8.6 Hz, H-5); 7.97 (1H, d, *J_meta_* = 2.0 Hz, H-7); 8.78 (1H, t, *J_NH-H_* = 5.6 Hz, CONH) ppm. ^13^C-NMR (100 MHz, DMSO-*d*_6_) δ 42.4 (NHCH_2_Ph); 44.7 (NHCH_2_CO); 103.5 (C-7); 113.5 (C-6); 118.5 (C-4); 127.15 (2C, C-2′, C6′); 127.11 (C-4′), 128.2 (2C, C-3′, C-5′); 134.3 (C-1′); 138.7 (C-5); 141.9 (C-7a); 149.7 (C-3a); 159.6 (C-2); 166.1 (C=O) ppm. MS (FAB): *m*/*z* 326 (M^+^H^+1^).

*2-(2-Amino-5(6)-nitro-1H-benzimidazol-1-yl)-N-phenylacetamide* (**2**). Yield: 80.1%; m.p. 285–288°C; Yellow solid, ^1^H-NMR and ^13^C-NMR indicate a 60:40 mixture of regioisomers.

*60% Regioisomer of 2-(2-amino-6-nitro-1H-benzimidazol-1-yl)-N-phenylacetamide* (**2a**).^1^H-NMR (400 MHz, DMSO-*d*_6_) δ 5.05 (2H, s, NHCH_2_CO); 7.06 (1H, dd, *J_orto_* = 7.2 Hz, *J_orto_* = 7.2 Hz, H-4′); 7.22 (2H, d, *J_orto_* = 9.2 Hz, H-2′, H-6′); 7.32 (2H, d, *J_orto_* = 8.0 Hz, H-3′, H-5′); 7.61 (2H, br, NH); 7.86 (1H, d, *J_orto_* = 8.0 Hz, H-6); 7.96 (1H, d, *J_orto_* = 8.0 Hz, H-7); 8.11 (1H, s, H-4); 10.52 (1H, br, CONH) ppm. ^13^C-NMR (100 MHz, DMSO-*d*_6_) δ 45.2 (NHCH_2_CO); 103.9 (C-7); 113.5 (C-6); 118.2 (C-4); 123.5 (C-4′); 127.2 (2C, C-2′, C-6′); 128.2 (2C, C-3′, C-5′); 138.8 (C-1′); 140.1 (C-5); 142.1 (C-7a); 142.8 (C-3a); 158.1 (C-2); 165.9 (C=O) ppm.

*40% Regioisomer of 2-(2-amino-5-nitro-1H-benzimidazol-1-yl)-N-phenylacetamide* (**2b**). ^1^H-NMR (400 MHz, DMSO-*d*_6_) δ 5.01 (2H, s, NHCH_2_CO); 7.06 (1H, dd, *J_orto_* = 7.2 Hz, *J_orto_* = 7.2 Hz, H-4′); 7.22 (2H, d, *J_orto_* = 9.2 Hz, H-2′, H-6′); 7.32 (2H, d, *J_orto_* = 8.0 Hz, H-3′, H-5′); 7.63 (br, 2H, NH); 7.86 (d, *J_orto_* = 8.8 Hz, 1H, H-4); 7.96 (1H, d, *J_orto_* = 8.0 Hz, H-5); 8.32 (s, 1H, H-7); 10.52 (1H, br, CONH) ppm. ^13^C-NMR (100 MHz, DMSO-*d*_6_) δ 45.6 (NHCH_2_CO); 107.4 (C-7); 109.4 (C-6); 114.9 (C-4); 119.1 (2C, C-2′, C-6′); 123.5 (C-4′). 128.9 (2C, C-3′, C-5′); 138.8 (C-1′); 140.5 (C-5); 142.1 (C-7a); 142.9 (C-3a); 158.4 (C-2); 164.9 (C=O) ppm. MS (FAB): *m*/*z* 312 (M^+^H^+1^).

*2-(2-Amino-5(6)-nitro-1H-benzimidazol-1-yl)-N-(4-fluorophenyl) acetamide* (**3**). Yield: 72.0%; m.p. 313.0–315.0 °C; Yellow solid, ^1^H-NMR and ^13^C-NMR indicate a 70:30 mixture of regioisomers.

*70% Regioisomer of 2-(2-amino-6-nitro-1H-benzimidazol-1-yl)-N-(4-fluorophenyl) acetamide* (**3a**). ^1^H-NMR (400 MHz, DMSO-*d*_6_) δ 4.98 (2H, s, NHCH_2_CO); 7.08 (2H, br, NH); 7.17 (2H, dd, *J_orto_* = 8.8 Hz, *J_orto_* = 8.8 Hz, H-3′, H5′); 7.30 (1H, d, *J_orto_ =* 8.8 Hz, H-7); 7.62 (2H, dd, *J_orto_ =* 5.2 Hz, *J_orto_ =* 8.8 Hz, H-2′, H6′); 7.87 (1H, dd, *J_meta_* = 2.4 Hz, *J_orto_ =* 8.8 Hz, H-6); 7.95 (1H, d, *J_meta_ =* 2.8 Hz, H-4); 10.47 (1H, s, CONH) ppm. ^13^C-NMR (100 MHz, DMSO-*d*_6_) δ 45.3 (NHCH_2_CO); 107.2 (C-7); 109.3 (C-6); 114.7 (C-4); 115.3 (d, *J_C-F_* = 56.2 Hz, 2C, C-3′, C-5′); 120.7 (d, *J_C-F_* = 7.6 Hz, 2C, C-2′, C-6′); 135.0 (C-1′); 140.3 (C-5); 141.9 (C-7a); 142.8 (C-3a); 158.1 (C-2); 159.3 (d, *J_C-F_* = 56.2 Hz, C-4′); 164.6 (C=O) ppm.

*30% Regioisomer of 2-(2-amino-5-nitro-1H-benzimidazol-1-yl)-N-(4-fluorophenyl) acetamide* (**3b**). ^1^H-NMR (400 MHz, DMSO-*d*_6_) δ 5.02 (2H, s, NHCH_2_CO); 7.33 (2H, br, NH); 7.17 (2H, dd, *J_orto_ =* 8.8 Hz, *J_orto_ =* 8.8 Hz, H-3′, H5′); 7.21 (1H, d, *J_orto_ =* 8.8 Hz, H-4); 7.62 (2H, dd, *J_meta_ =* 5.2 Hz, *J_orto_ =* 8.8 Hz, H-2′, H6′); 7.87 (1H, dd, *J_meta_ =* 2.4 Hz, *J_orto_ =* 8.8 Hz, H-5); 8.12 (1H, d, *J_meta_ =* 2.4 Hz, H-7); 10.47 (1H, s, CONH) ppm. ^13^C-NMR (100 MHz, DMSO-*d*_6_) δ 45.1 (NHCH_2_CO); 103.7 (C-7); 113.3 (C-6); 118.0 (C-4); 120.7 (d, *J_C-F_* = 7.6 Hz, 2C, C-2′, C-6′); 159.3 (d, *J_C-F_* = 56.2 Hz, C-4′); 115.3 (d, *J_C-F_* = 56.2 Hz, 2C, C-3′, C-5′); 134.6 (C-1′); 138.8 (C-5); 141.9 (C-7a); 149.6 (C-3a); 156.7 (C-2); 164.7 (C=O) ppm. MS (FAB): *m*/*z* 330 (M^+^H^+1^).

*2-(2-Amino-5(6)-nitro-1H-benzimidazol-1-yl)-N-(4-chlorophenyl) acetamide* (**4**). Yield: 92.1%; m.p. 294.0–296.0 °C; Yellow solid, ^1^H-NMR and ^13^C-NMR indicate 69:31 mixture of regioisomers.

*69% Regioisomer of 2-(2-amino-6-nitro-1H-benzimidazol-1-yl)-N-(4-chlorophenyl) acetamide* (**4a**). ^1^H-NMR (400 MHz, DMSO-*d*_6_) δ 5.04 (2H, s, NHCH_2_CO); 7.32 (2H, br, NH); 7.37 (1H, d, *J_orto_ =* 8.8 Hz, H-7); 7.63 (2H, d, *J_meta_ =* 2.4 Hz, *J_orto_ =* 5.2 Hz, H-3′, H-5′); 7.65 (2H, d, *J_meta_ =* 2.0 Hz, *J_orto_ =* 5.2 Hz, H-2′, H-6′); 7.96 (1H, d, *J_meta_ =* 2.8 Hz, *J_orto_ =* 8.8 Hz, H-6); 8.12 (1H, d, *J_meta_ =* 2.0 Hz, H-4); 10.56 (s, 1H, CONH) ppm. ^13^C-NMR (100 MHz, DMSO-*d*_6_) δ 45.2 (NHCH_2_CO); 103.9 (C-7); 113.4 (C-6); 118.1 (C-4); 120.5 (2C, C-2′,C-6′); 126.9 (C-4′); 128.7 (2C, C-3′,C-5′); 137.6 (C-1′); 138.8 (C-5); 141.9 (C-7a); 149.6 (C-3a); 159.7 (C-2); 165.1 (C=O) ppm.

*31% Regiisomer of 2-(2-amino-5-nitro-1H-benzimidazol-1-yl)-N-(4-chlorophenyl) acetamide* (**4b**). ^1^H-NMR (400 MHz, DMSO-*d*_6_) δ 4.99 (2H, s, NHCH_2_CO); 7.06 (2H, br, NH); 7.21 (1H, d, *J_orto_ =* 8.8 Hz, H-4); 7.63 (2H, d, *J_meta_ =* 2.4 Hz, *J_orto_ =* 5.2 Hz, H-3′, H-5′); 7.65 (2H, d, *J_meta_ =* 2.0 Hz, *J_orto_ =* 5.2 Hz, H-2′, H-6′); 7.86 (1H, d, *J_meta_ =* 2.0 Hz, *J_orto_ =* 8.8 Hz, H-5); 7.96 (1H, d, *J_meta_ =* 2.0 Hz, H-7); 10.59 (s, 1H, CONH) ppm. ^13^C-NMR (100 MHz, DMSO-*d*_6_) δ 45.3 (NHCH_2_CO); 107.3 (C-7); 109.3 (C-6); 114.8 (C-4); 120.5 (2C, C-2′, C-6′); 126.9 (C-4′); 128.7 (2C, C-3′, C-5′); 134.6 (C-1′); 140.3 (C-5); 141.9 (C-7a); 142.7 (C-3a); 158.6 (C-2); 164.9 (C=O) ppm. MS (FAB): *m*/*z* 346 (M^+^H^+1^).

*2-(2-Amino-5(6)-nitro-1H-benzimidazol-1-yl)-N-(4-cyanophenyl) acetamide* (**5**). Yield: 81.5%; m.p. 254.0–256.7 °C; Yellow solid, ^1^H-NMR and ^13^C-NMR indicate 57:43 mixture of regioisomers.

*57% Regioisomer of 2-(2-amino-6-nitro-1H-benzimidazol-1-yl)-N-(4-cyanophenyl) acetamide* (**5a**). ^1^H-NMR (400 MHz, DMSO-*d*_6_) δ 5.08 (2H, s, NHCH_2_CO); 7.07 (2H, br, NH); 7.22 (1H, d, *J_orto_ =* 8.8 Hz, H-7); 7.79 (4H, s, H-2′, H-3′, H-5′, H-6′); 7.85 (1H, d, *J_orto_ =* 8.8 Hz, H-6); 7.94 (1H, d, *J_meta_ =* 2.0 Hz, H-4); 10.84 (1H, s, CONH) ppm. ^13^C-NMR (100 MHz, DMSO-*d*_6_) δ 45.3 (NHCH_2_CO); 103.9 (C-7); 105.2 (C-4′); 113.4 (C-6); 118.1 (C-4); 118.9 (CN); 119.2 (2C, C-2′,C-6′); 133.4 (2C, C-3′, C-5′); 134.6 (C-1′); 138.9 (C-5); 142.8 (C-7a); 149.6 (C-3a); 159.6 (C-2); 165.8 (C=O) ppm.

*43% Regioisomer of 2-(2-amino-5-nitro-1H-benzimidazol-1-yl)-N-(4-cyanophenyl) acetamide* (**5b**). ^1^H-NMR (400 MHz, DMSO-*d*_6_) δ 5.03 (2H, s, NHCH_2_CO); 7.32 (2H, br, NH); 7.33 (1H, d, *J_orto_ =* 8.8 Hz, H-4); 7.79 (4H, s, H-2′, H-3′, H-5′, H-6′); 7.85 (1H, d, *J_orto_ =* 8.8 Hz, H-5); 7.97 (1H, d, *J_meta_ =* 2.0 Hz, H-7); 10.87 (1H, s, CONH) ppm. ^13^C-NMR (100 MHz, DMSO-*d*_6_) δ 45.5 (NHCH_2_CO); 103.3 (C-7); 105.2 (C-4′); 109.4 (C-6); 114.8 (C-4); 118.9 (CN); 119.2 (2C, C-2′, C-6′); 133.4 (2C, C-3′, C-5′); 134.6 (C-1′); 140.3 (C-5); 142.7 (C-7a); 142.0 (C-3a); 158.1 (C-2); 165.7 (C=O) ppm. MS (FAB): *m*/*z* 337 (M^+^H^+1^).

*2-(2-Amino-5(6)-nitro-1H-benzimidazol-1-yl)-N-(4-nitrophenyl) acetamide* (**6**). Yield: 79.8%; m.p. 309.0–312.0 °C; Yellow solid, ^1^H-NMR and ^13^C-NMR indicates a 59:41mixture of reigoisomers.

*59% Regioisomer of 2-(2-amino-6-nitro-1H-benzimidazol-1-yl)-N-(4-nitrophenyl) acetamide* (**6a**). ^1^H-NMR (400 MHz, DMSO-*d*_6_) δ 5.11 (2H, s, NHCH_2_CO); 7.34 (2H, br, NH); 7.36 (1H, d, *J_orto_ =* 8.8 Hz, H-7); 7.85 (2H, dd, *J_meta_ =* 2.8 Hz, *J_orto_ =* 5.2 Hz, H-2′, H-6′); 7.86 (2H, dd, *J_meta_ =* 2.4 Hz, *J_orto_ =* 8.0 Hz, H-3′, H-5′); 8.19 (1H, d, *J_meta_ =* 2.0 Hz, H-4); 8.25 (1H, d, *J_orto_ =* 8.4 Hz, H-6); 11.04 (1H, s, CONH) ppm. ^13^C-NMR (100 MHz, DMSO-*d*_6_) δ 45.4 (NHCH_2_CO); 104.0 (C-7); 144.9 (C-4′); 113.4 (C-6); 118.1 (C-4); 118.7 (2C, C-2′, C-6′); 125.1 (2C, C-3′, C-5′); 134.6 (C-1′); 138.9 (C-5); 142.2 (C-7a); 149.6 (C-3a); 159.6 (C-2); 166.0 (C=O) ppm.

*41% Regioisomer compound 2-(2-amino-5-nitro-1H-benzimidazol-1-yl)-N-(4-nitrophenyl) acetamide* (**6b**). ^1^H-NMR (400 MHz, DMSO-*d*_6_) δ 5.06 (2H, s, NHCH_2_CO); 7.09 (2H, br, NH); 7.22 (d, *J_orto_ =* 8.8 Hz, 1H, H-4); 7.83 (1H, d, *J_meta_ =* 2.4 Hz, H-7); 7.95 (2H, dd, *J_meta_ =* 2.8 Hz, *J_orto_ =* 5.2 Hz, H-2′, H-6′); 7.96 (2H, dd, *J_meta_ =* 2.4 Hz, *J_orto_ =* 8.0 Hz, H-3′, H-5′); 8.25 (1H, d, *J_orto_ =* 8.4 Hz, H-5); 11.01 (1H, s, CONH) ppm. ^13^C-NMR (100 MHz, DMSO-*d*_6_) δ 45.6 (NHCH_2_CO); 107.4 (C-7); 144.7 (C-4′); 109.4 (C-6); 114.8 (C-4); 118.7 (2C, C-2′, C-6′); 125.1 (2C, C-3′, C-5′); 134.6 (C-1′); 140.3 (C-5); 142.0 (C-7a); 142.7 (C-3a); 158.1 (C-2); 165.8 (C=O) ppm. MS (FAB): *m*/*z* 357 (M^+^H^+1^).

*2-(2-Amino-5(6)-nitro-1H-benzimidazol-1-yl)-N-(2,6-dichlorophenyl) acetamide* (**7**). Yield: 61.4%; m.p. 303.0–306.0 °C; Yellow solid, ^1^H-NMR and ^13^C-NMR indicate a 55:45 mixture of reigoisomers.

*55% Regioisomer of 2-(2-amino-6-nitro-1H-benzimidazol-1-yl)-N-(2,6-dichlorophenyl) acetamide* (**7a**). ^1^H-NMR (400 MHz, DMSO-*d*_6_) δ 5.11 (2H, s, NHCH_2_CO); 7.21 (1H, d, *J_orto_ =* 8.4 Hz, H-7); 7.36 (2H, dd, *J_meta_ =* 3.8 Hz, *J_orto_ =* 8.4 Hz, H-3′, H-5′); 7.41 (2H, br, NH); 7.54 (1H, d, *J_orto_ =* 8.0 Hz, H-4′); 7.97 (1H, d, *J_meta_ =* 2.6 Hz, *J_orto_ =* 8.6 Hz, H-6); 8.02 (1H, d, *J_meta_ =* 2.4 Hz, H-4); 10.45 (1H, s, CONH) ppm. ^13^C-NMR (100 MHz, DMSO-*d*_6_) δ 44.6 (NHCH_2_CO); 103.7 (C-7); 109.4 (C-6); 114.8 (C-4); 128.5 (2C, C-3′, C-5′); 129.5 (2C, C-2′, C-6′); 132.2 (C-1′); 133.4 (C-4′); 138.7 (C-5); 142.0 (C-7a); 142.9 (C-3a); 158.0 (C-2); 165.2 (C=O) ppm.

*45% Regioisomer of 2-(2-amino-5-nitro-1H-benzimidazol-1-yl)-N-(2,6-dichlorophenyl)acetamide* (**7b**). ^1^H-NMR (400 MHz, DMSO-*d*_6_) δ 5.08 (2H, s, NHCH_2_CO); 7.12 (2H, br, NH); 7.21 (1H, d, *J_orto_ =* 8.8 Hz, H-4); 7.34 (2H, dd, *J_meta_ =* 3.6 Hz, *J_orto_ =* 8.4 Hz, H-3′, H-5′); 7.55 (1H, d, *J_orto_ =* 8.0 Hz, H-4′); 7.91 (1H, d, *J_meta_ =* 2.0 Hz, *J_orto_ =* 8.8 Hz, H-5); 7.94 (1H, d, *J_meta_ =* 2.4 Hz, H-7); 10.45 (1H, s, CONH) ppm. ^13^C-NMR (100 MHz, DMSO-*d*_6_) δ 44.8 (NHCH_2_CO); 107.1 (C-7); 113.4 (C-6); 118.24 (C-4); 128.5 (2C, C-3′, C-5′); 129.5 (2C, C-2′, C-6′); 134.1 (C-1′); 133.4 (C-4′); 140.0 (C-5); 142.0 (C-7a); 149.8 (C-3a); 159.6 (C-2); 165.3 (C=O) ppm. MS (FAB): *m*/*z* 381 (M^+^H^+1^).

*2-(2-Amino-5(6)-nitro-2,3-dihydro-1H-benzimidazol-1-yl)-N-[3-(trifluoromethyl)phenyl]acetamide* (**8**). Yield: 60.7%; m.p. 282.0–284.0 °C; Yellow solid, ^1^H-NMR and ^13^C-NMR indicate 61:39 mixture of regioisomers.

*61% Regioisomer of 2-(2-amino-6-nitro-2,3-dihydro-1H-benzimidazol-1-yl)-N-[3-(trifluoromethyl)phenyl]* *acetamide* (**8a**). ^1^H-NMR (400 MHz, DMSO-*d*_6_) δ 5.07 (2H, s, NHCH_2_CO); 7.32 (2H, br, NH); 7.22 (1H, d, *J_orto_ =* 8.4 Hz, H-7); 8.10 (1H, d, *J_meta_ =* 2.8 Hz, H-2′); 7.85 (2H, dd, *J_meta_ =* 2.8 Hz, *J_orto_ =* 5.2 Hz, H-6′); 7.86 (2H, dd, *J_meta_ =* 2.4 Hz, *J_orto_ =* 8.0 Hz, H-4′, H-5′); 8.15 (1H, d, *J_meta_ =* 2.4 Hz, H-4); 7.96 (1H, dd, *J_meta_ =* 2.2 Hz, *J_orto_ =* 8.6 Hz, H-6); 10.73 (1H, s, CONH) ppm. ^13^C-NMR (100 MHz, DMSO-*d*_6_) δ 45.2 (NHCH_2_CO); 103.9 (C-7); 109.4 (C-6); 115.0 (C-2′); 119.8 (C-4′); 118.1 (C-4); 118.1 (C-6′); 122.5 (q, *J_C-F_* = 277.8 Hz, CF_3_); 129.5 (q, *J_C-F_* = 33.4 Hz, C-3′); 130.1 (C-5′); 134.6 (C-1′); 138.9 (C-5); 141.9 (C-7a); 149.7 (C-3a); 159.7 (C-2); 165.6 (C=O) ppm.

*39% Regiosiomer of 2-(2-amino-5-nitro-2,3-dihydro-1H-benzimidazol-1-yl)-N-[3-(trifluoromethyl)phenyl]* *acetamide* (**8b**). ^1^H-NMR (400 MHz, DMSO-*d*_6_) δ 5.06 (2H, s, NHCH_2_CO); 7.09 (2H, br, NH); 7.22 (1H, d, *J_orto_ =* 8.8 Hz, H-4); 7.83 (1H, d, *J_meta_ =* 2.4 Hz, H-7); 7.95 (2H, dd, *J_meta_ =* 2.8 Hz, *J_orto_ =* 5.2 Hz, H-2′, H-6′); 7.96 (2H, dd, *J_meta_ =* 2.4 Hz, *J_orto_ =* 8.0 Hz, H-4′, H-5′); 8.25 (1H, d, *J_orto_ =* 8.4 Hz, H-5); 11.01 (1H, s, CONH) ppm. ^13^C-NMR (100 MHz, DMSO-*d*_6_) δ 45.3 (NHCH_2_CO); 107.3 (C-7); 113.4 (C-6); 115.0 (C-2′); 119.8 (C-4′); 114.8 (C-4); 118.1 (C-6′); 122.5 (q, *J_C-F_* = 277.8 Hz, CF_3_); 129.5 (q, *J_C-F_* = 33.4 Hz, C-3′); 140.3 (C-5′); 134.6 (C-1′); 140.3 (C-5); 141.9 (C-7a); 142.8 (C-3a); 158.1 (C-2); 165.4 (C=O) ppm. MS (FAB): *m*/*z* 381 (M^+^H^+1^).

NMR spectra of compounds **1**–**8** can be found in Supplementary Materials.

### 4.3. Biological Assays

*G. intestinalis* strain IMSS:0696:1 was cultured in a TYI-S-33 modified medium, supplemented with 10% calf serum and bovine bile. The *T. vaginalis* strain GT3 and *E. histolytica* HM1-IMSS were cultured in TYI-S-33 medium, supplemented with 10% bovine serum. In vitro susceptibility assays were performed using a method described previously [26,45]. Briefly, 4 × 10^4^ trophozoites of *G. intestinalis* or *T. vaginalis* or *E. histolytica* were incubated for 48 h at 37 °C with increasing concentrations of the synthesized benznidazole and metronidazole compounds. Trophozoites incubated in a culture medium with DMSO were used as the negative control in the experiments. After incubation, trophozoites were washed and subcultured for another 48 h in fresh medium alone. At the end of this period, trophozoites were counted and the 50% inhibitory concentration (IC_50_) was calculated by Probit analysis. Experiments were carried out in triplicate and repeated at least twice.

### 4.4. Computational Chemistry

All calculations of the NMR shielding constants have been performed using gauge-including atomic orbitals (GIAO) [38] in DMSO solvent. We employed the nwchem program package (version 6.5) [39]. Geometry optimizations were carried out at the DFT level with the B3LYP exchange-correlation functional [40], with Def2-TZVP [41] basis set. Harmonic vibrational frequencies are also analyzed at the same level to characterize the nature of the stationary molecules.

## 5. Conclusions

We synthesized a series of benzimidazole derivatives obtaining inseparables mixtures of regioisomers, with the 1,6-substituted compound as the predominant one. Products were characterized by ^1^H- and ^13^C-NMR, where we corroborated that the predominant regioisomer was substituted at the 6-position. In addition, computational calculations of the regioisomer mixtures conformed the match between the predicted chemical displacements with the experimental findings, confirming the substitution of the nitro group in position 6 as the predominant regioisomer. The mixture of regioisomers demonstrated interesting activity against two intestinal parasites: *Giardia* and *Trichomonas*. Compound **8** obtained an IC_50_ of 3.84 μM against *G. intestinalis*, and an IC_50_ of 5.62 μM against *T. vaginalis.* This compound bears a –CF_3_ substituted in the *meta* position of the aryl acetamide. All compounds show good antiprotozoal profiles against *G. intestinalis* and *T. vaginalis* in comparison with benznidazole.

## Figures and Tables

**Table 1:** Calculated activity and toxicity in silico profiles for compounds **1**–**8** and antiprotozoal choice drugs.

Compound	Antiparasitic Effect (*Trichomonas vaginalis*)		LD_50_ (mg/Kg)			
			Mouse		Rat	
	*Pa*	*Pi*	ip	p.o.	ip	po
**1**	0.313	0.023	300	1300	430	1200
**2**	0.319	0.020	460	1600	330	830
**3**	0.250	0.065	490	830	530	670
**4**	0.304	0.027	490	840	460	970
**5**	0.201	0.122	380	960	800	320
**6**	0.324	0.018	430	1200	1200	670
**7**	0.292	0.033	290	920	280	900
**8**	0.201	0.122	280	1100	440	750
**10**	0.333	0.015	150	1200	480	620
**Bnz**	0.705	0.002	1100	280	1300	1100

Pa = Probability of activity Pi = Probability of inactivity ip = Intraperitoneal po = Oral.

**Table 2:** Physicochemical properties, and in vitro antiprotozoal bioactivity benznidazole analogues **1**–**8**.

Cmpd	m.p. (°C)	Reaction Time (h)	Yield (%)	MS FAB (+)		IC_50_ (μM)		
					Reigoisomers Mixture Ratio	*T. vaginalis*	*G. lamblia*	*E. histolytica*
**1**	279.0–282.0	5.0	90.1	326	75:25	11.34	11.26	18.43
**2**	285.0–287.8	5.0	80.1	297	60:40	26.76	10.44	46.16
**3**	313.0–315.0	7.0	72.0	330	70:30	15.61	16.47	26.14
**4**	294.0–296.0	12.0	92.1	345	69:31	14.66	15.26	28.28
**5**	286.0–289.0	8.0	81.5	337	57:43	11.16	14.45	21.07
**6**	309.0–312.0	8.0	79.8	357	59:41	7.09	16.52	13.23
**7**	303.0–306.0	18.0	61.4	381	55:45	5.92	9.20	34.01
**8**	282.0–284.0	18.0	60.7	380	61:39	5.62	3.84	45.60
**10**	192.0–194.0	5.0	94.1	179	50:50 *	22.12	28.39	46.80
Bnz	---	---	---	---	---	18.62	22.58	4.27

ND = not determinate; * Tautomeric equilibrium.

**Table 3:**
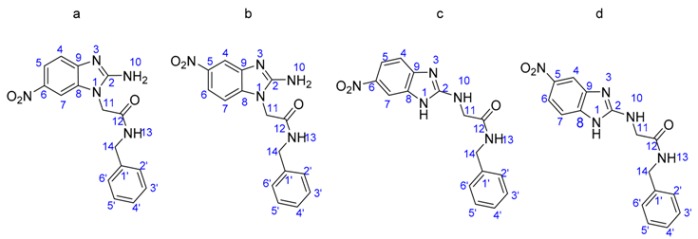
NMR predictions for reigoisomers and tautomer compounds.

Label	Experimental (δ, DMSO-*d*_6_)				In Silico (δ, DMSO-*d*_6_)							
	^1^H-NMR		^13^C-NMR		^1^H-NMR				^13^C-NMR			
	a	b	a	b	a	b	c	d	a	b	c	d
**1**	---	---	---	---	---	---	6.89	7.13	---	---	---	---
**2**	---		158.05	159.58	---	---	---	---	163.58	166.01	161.77	158.55
**3**	---	---	---	---	---	---	---	---	---	---	---	---
**4**	7.93	7.97	114.67	118.04	6.75	7.52	7.54	7.18	120.50	121.30	121.08	119.48
**5**	7.87	---	140.14	138.70	---	8.57	8.60	---	152.59	148.66	149.48	152.78
**6**	---	7.95	109.32	113.38	8.38	---	---	8.44	121.72	125.74	126.13	121.83
**7**	7.19	7.20	107.06	103.48	8.74	8.10	8.29	8.71	107.17	106.15	108.73	109.66
**8**	---	---	141.88	141.88	---	---	---	---	145.53	140.09	138.02	144.21
**9**	---	---	142.81	149.73	---	---	---	---	151.13	157.75	183.91	151.70
**10**	7.03	7.03	---	---	5.54	5.82	3.81	5.77	---	---	---	---
**11**	4.83	4.87	44.87	44.72	4.30	4.31	4.25	3.74	50.92	50.93	49.35	48.10
**12**	---	---	165.93	166.08	---	---	---	---	173.12	173.57	172.21	169.12
**13**	8.73	8.78	---	---	5.15	5.32	4.98	4.61	---	---	---	---
**14**	4.32	4.33	42.41	42.39	4.61	4.62	4.60	4.77	48.44	48.56	47.53	48.10
**1′**	---	---	138.77	134.31	---	---	---	---	145.57	144.75	147.96	147.03
**2′**	7.23–7.36		127.20	127.15	7.48	7.50	7.44	7.63	135.56	135.36	135.06	134.90
**3′**	7.23–7.36		127.20	128.19	7.64	7.63	7.75	7.69	134.15	134.59	134.90	134.12
**4′**	7.23–7.36		126.79	127.13	7.62	7.61	7.70	7.68	134.88	134.92	133.23	134.04
**5′**	7.23–7.36		127.20	128.19	7.63	7.62	7.63	7.74	134.30	134.11	134.47	134.53
**6′**	7.23–7.36		127.20	127.15	7.50	7.46	7.72	7.47	135.25	135.38	136.46	135.65

## Supplementary Materials

Supplementary materials are available online.
