# Supplementary material for: Design, Synthesis and Biological Evaluation of 2-(2-Amino-5(6)-nitro-1H-benzimidazol-1-yl)-N-arylacetamides as Antiprotozoal Agents
Source: Molecules. 2017 Apr 4;22(4):579. doi: 10.3390/molecules22040579 (PMC6154724; doi:10.3390/molecules22040579)
Supplement: Supplementary file 1 [file molecules-22-00579-s001.pdf]

Design, Synthesis and Biological Evaluation of 2-(2-Amino-5(6)-nitro-1*H*-benzimidazol-1-yl)-*N*-aryl-acetamides as Antiprotozoal Agents

Emanuel Hernández-Núñez [1*], Hugo Tlahuext [2], Rosa Moo-Puc [3], Diego Moreno[4], María Ortencia González-Díaz[5], and Gabriel Navarrete Vázquez [6,]

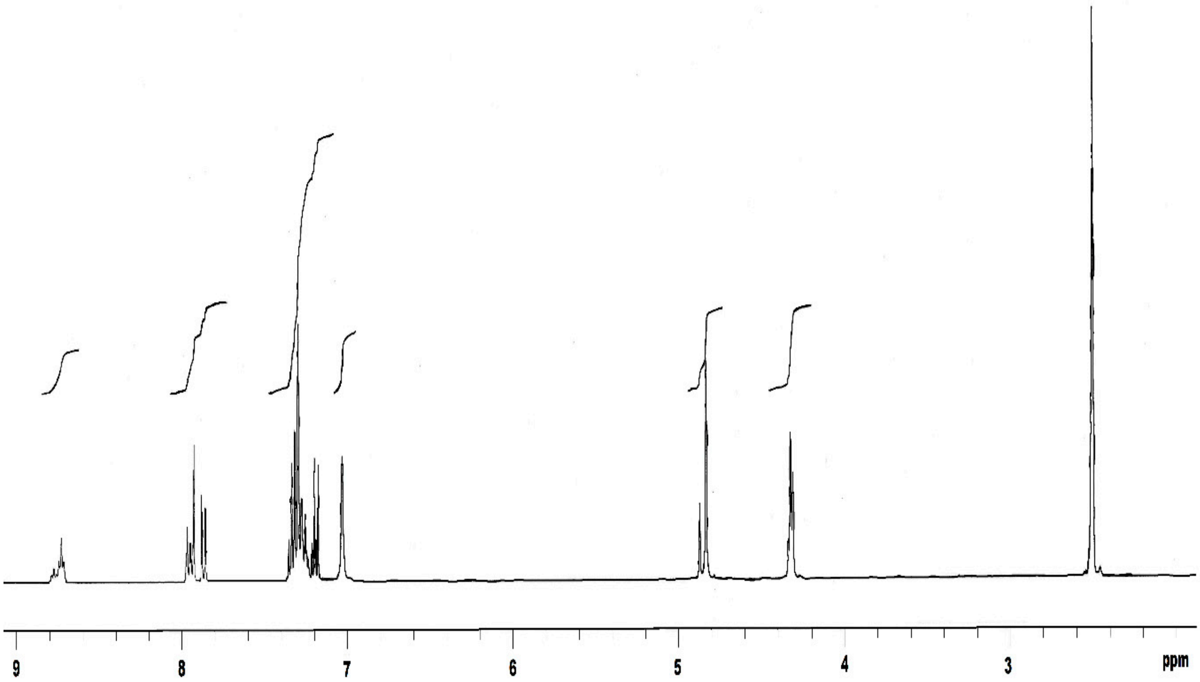

Figure S1. ^{1}H-NMR 2-(2-amino-5(6)-nitro-1*H*-benzimidazol-1-yl)-*N*-benzylacetamide (**1**).

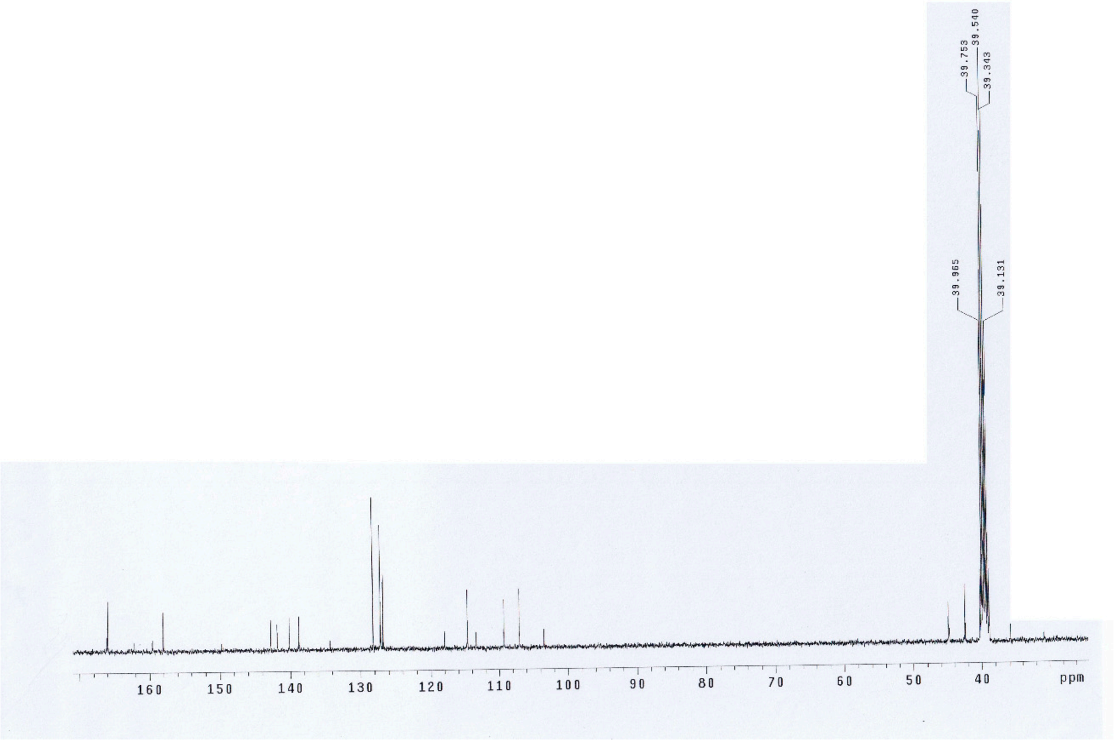

Figure S2. ^{13}C-NMR 2-(2-amino-5(6)-nitro-1*H*-benzimidazol-1-yl)-*N*-benzylacetamide (**1**).

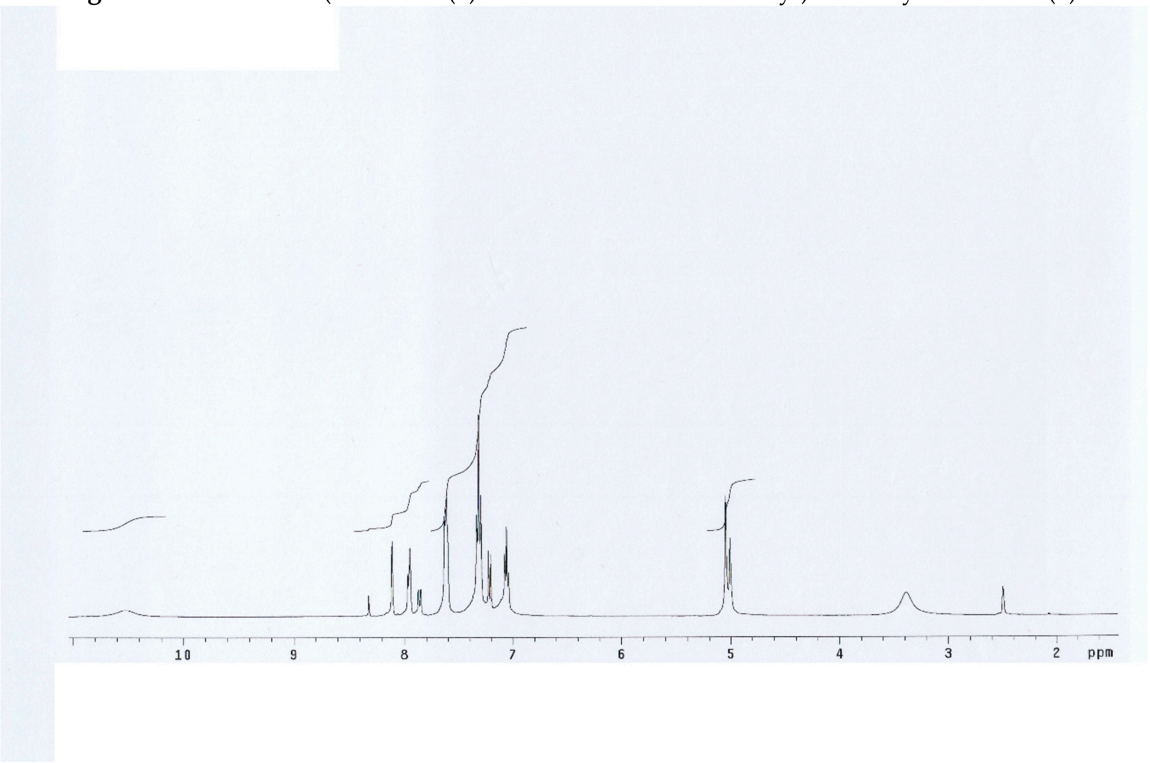

Figure S3. ^{1}H-NMR 2-(2-amino-5(6)-nitro-1*H*-benzimidazol-1-yl)-*N*-phenylacetamide (**2**).

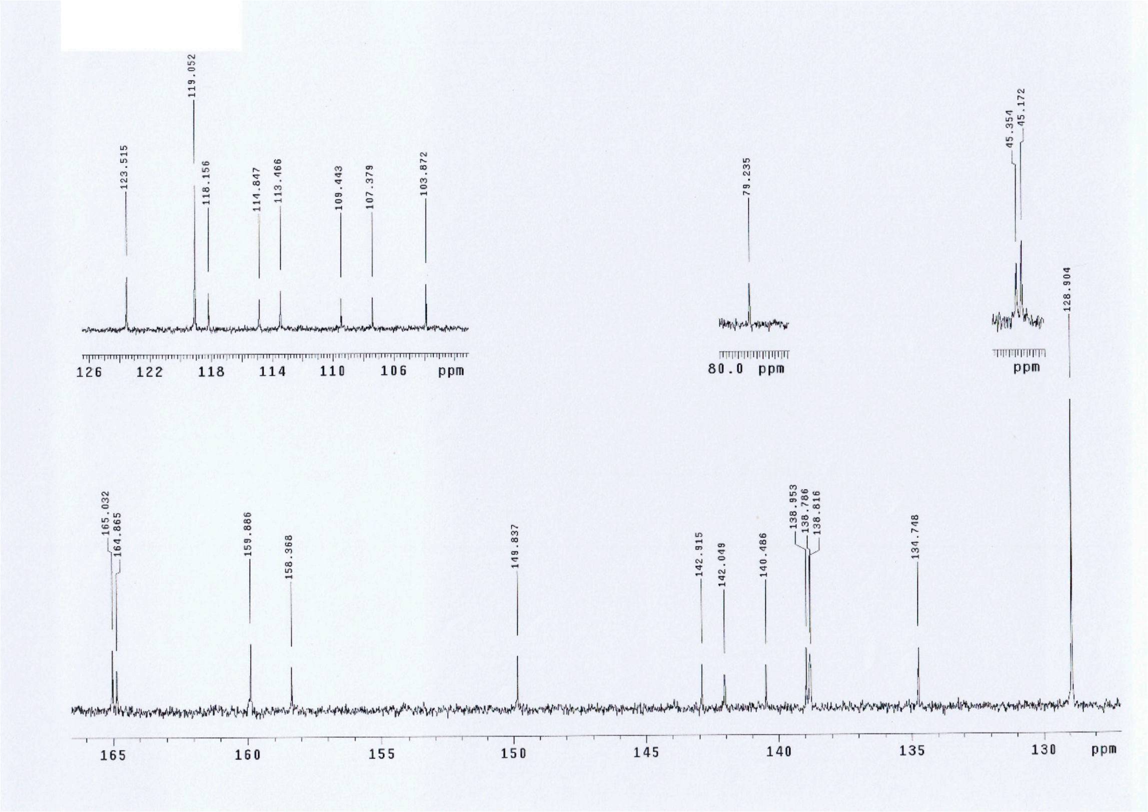

Figure S4. ^{13}C-NMR 2-(2-amino-5(6)-nitro-1*H*-benzimidazol-1-yl)-*N*-phenylacetamide (**2**).

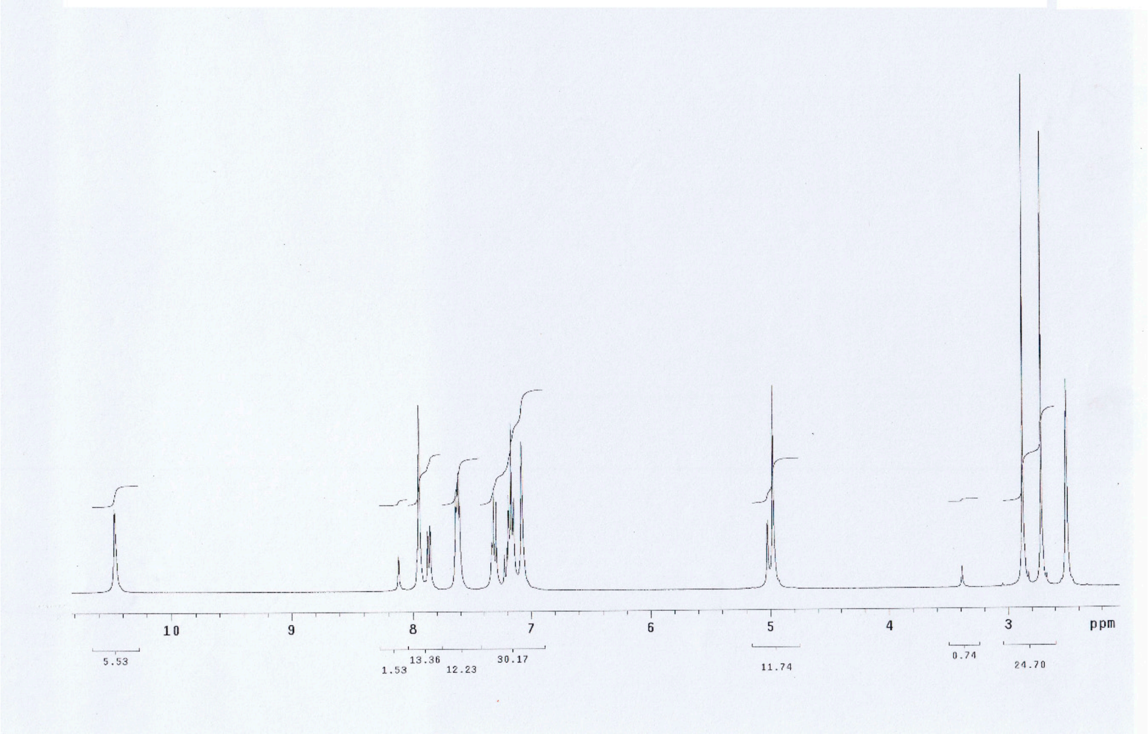

Figure S5. ^{1}H-NMR 2-(2-amino-5(6)-nitro-1*H*-benzimidazol-1-yl)-*N*-(4-fluorophenyl) acetamide (**3**).

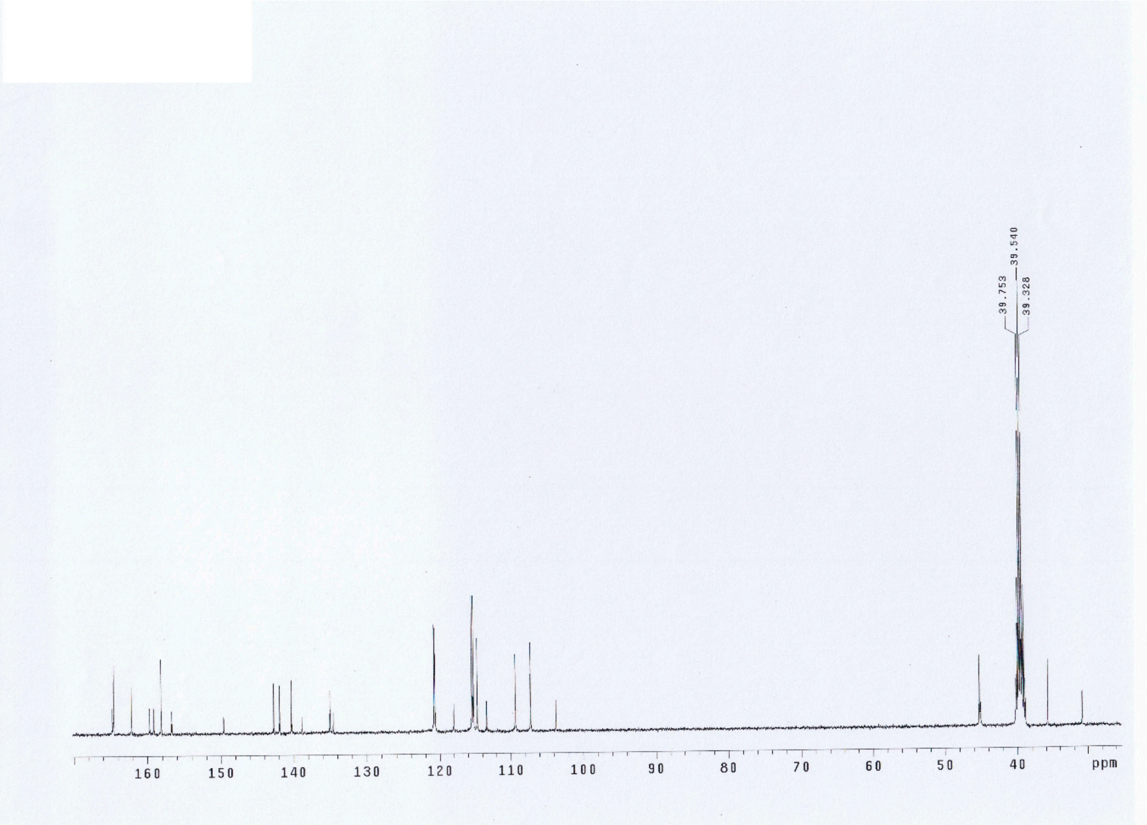

Figure S6. ^{13}C-NMR 2-(2-amino-5(6)-nitro-1*H*-benzimidazol-1-yl)-*N*-(4-fluorophenyl) acetamide (**3**).

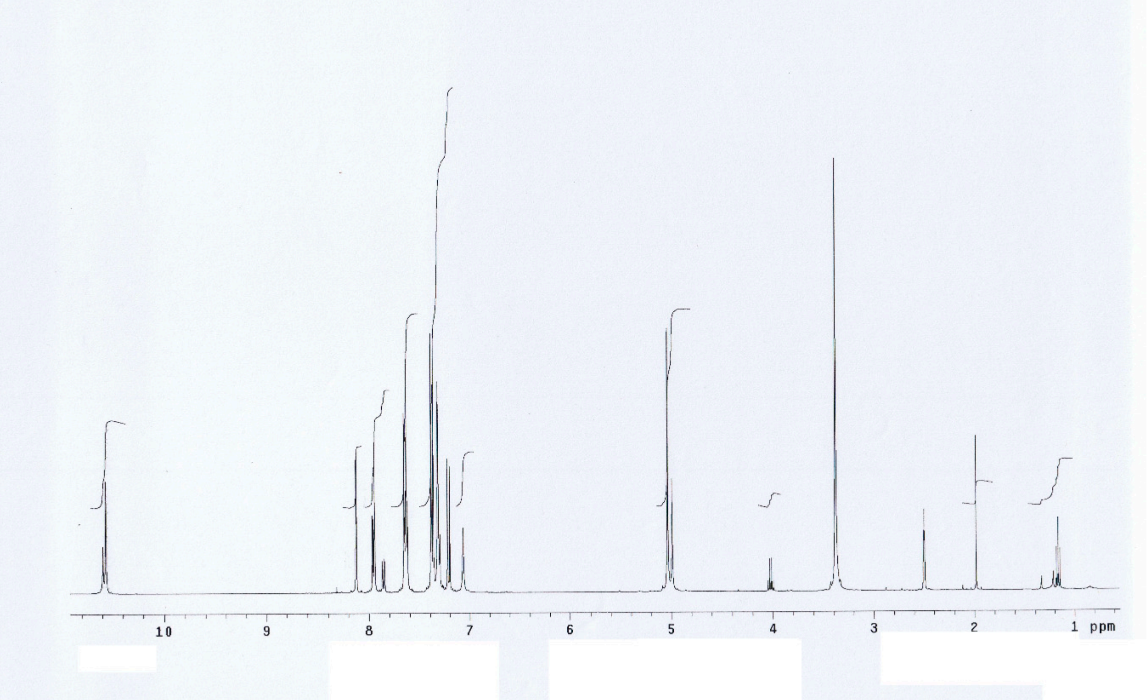

Figure S7. ^{1}H-NMR 2-(2-amino-5(6)-nitro-1*H*-benzimidazol-1-yl)-*N*-(4-chlorophenyl) acetamide (**4**).

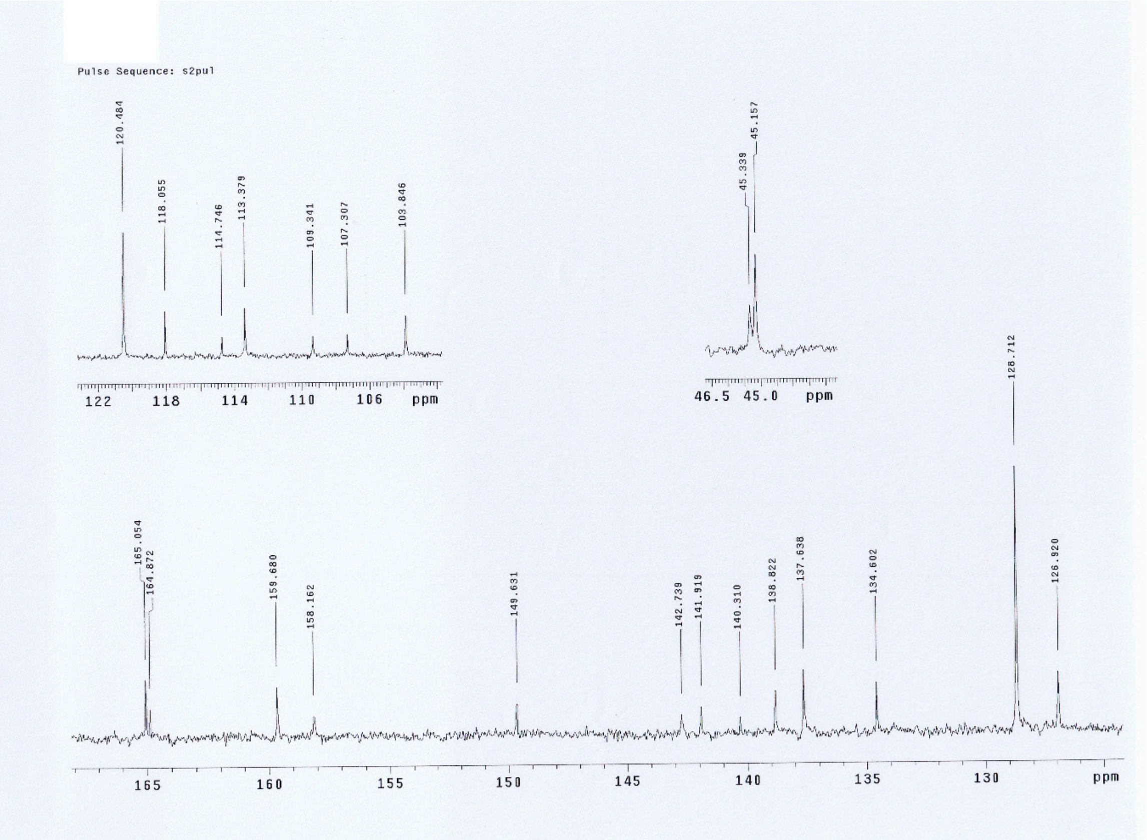

Figure S8. ^{13}C-NMR 2-(2-amino-5(6)-nitro-1*H*-benzimidazol-1-yl)-*N*-(4-chlorophenyl) acetamide (**4**).

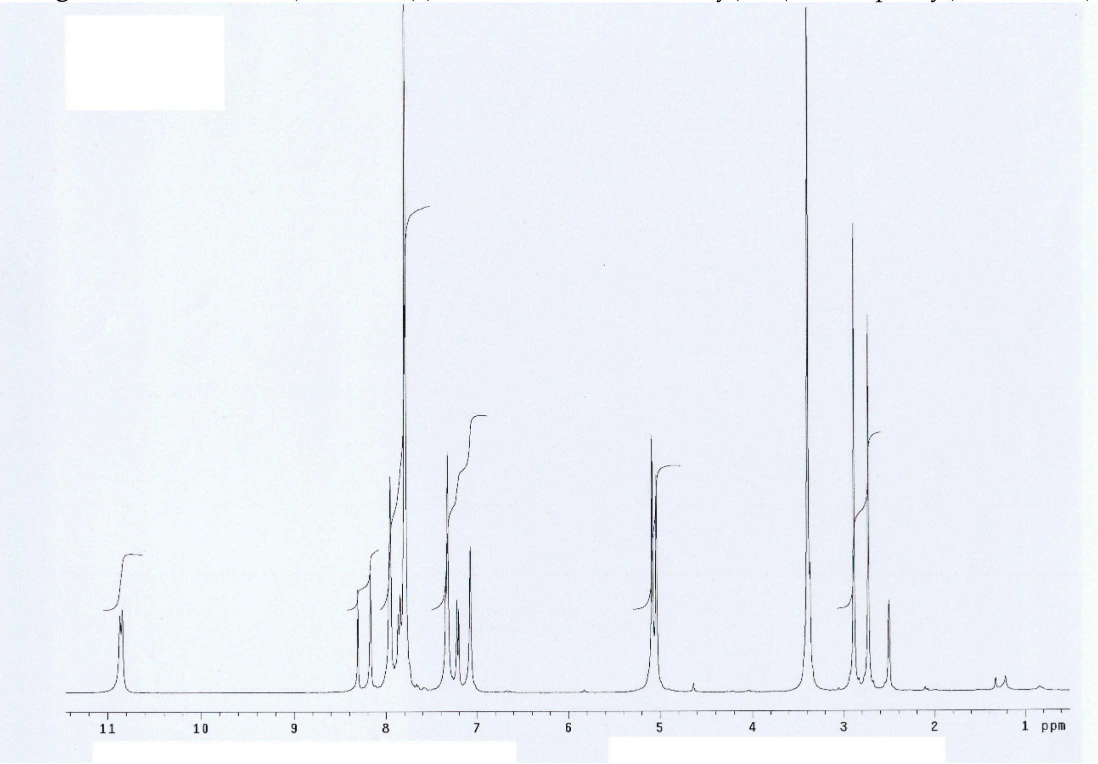

Figure S9. ^{1}H-NMR 2-(2-amino-5(6)-nitro-1*H*-benzimidazol-1-yl)-*N*-(4-cyanophenyl) acetamide (**5**).

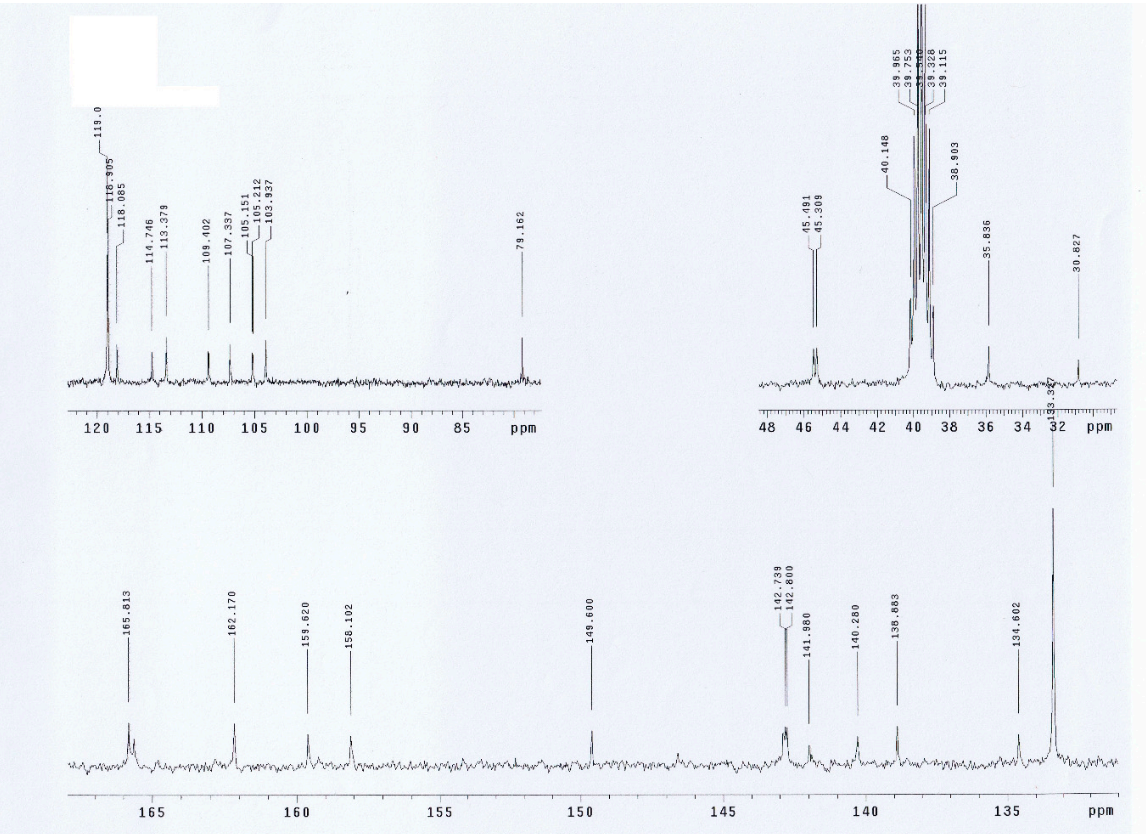

Figure S10. ^{13}C-NMR 2-(2-amino-5(6)-nitro-1*H*-benzimidazol-1-yl)-*N*-(4-cyanophenyl) acetamide (**5**).

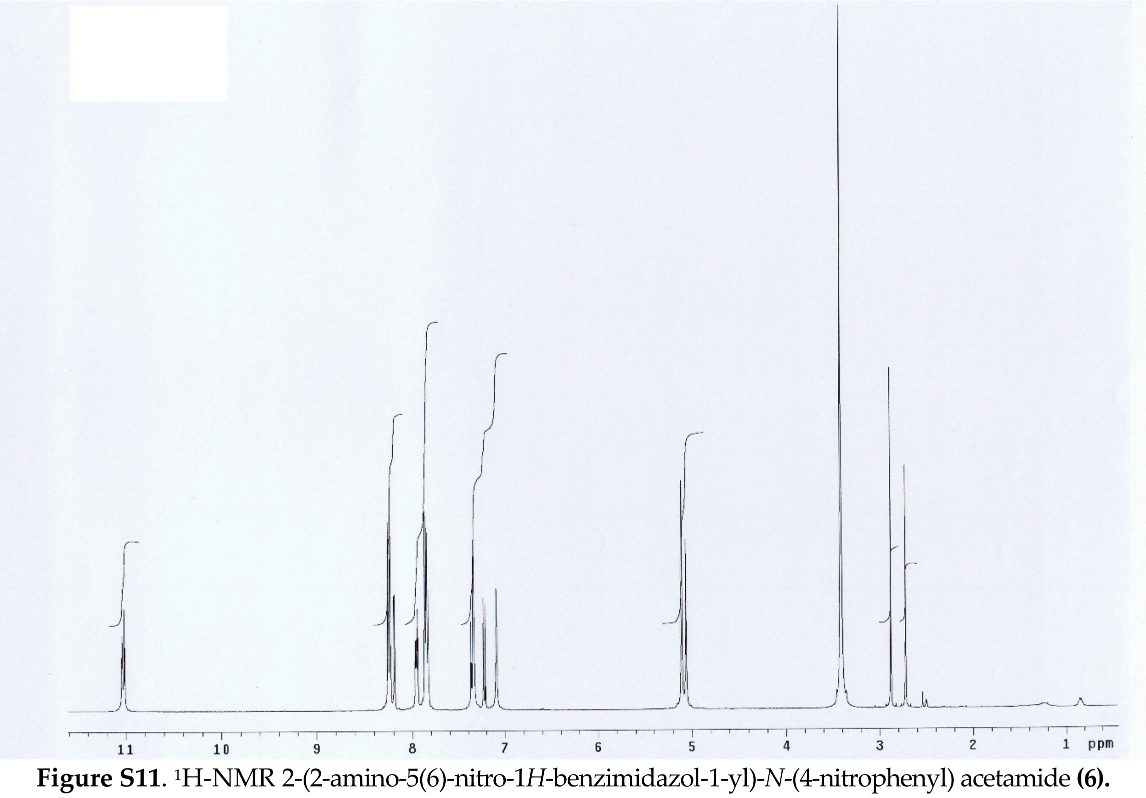

Figure S11. ^{1}H-NMR 2-(2-amino-5(6)-nitro-1*H*-benzimidazol-1-yl)-*N*-(4-nitrophenyl) acetamide **(6).**

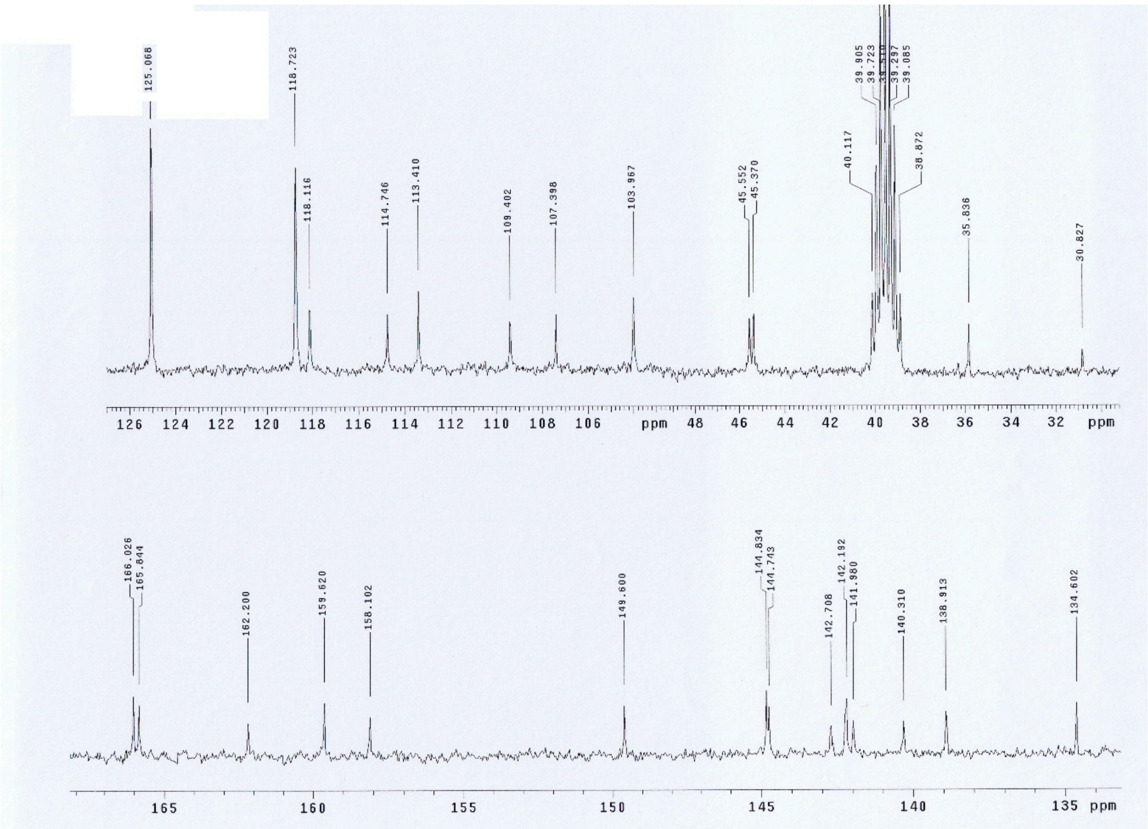

Figure S12. ^{13}C-NMR 2-(2-amino-5(6)-nitro-1*H*-benzimidazol-1-yl)-*N*-(4-nitrophenyl) acetamide **(6).**

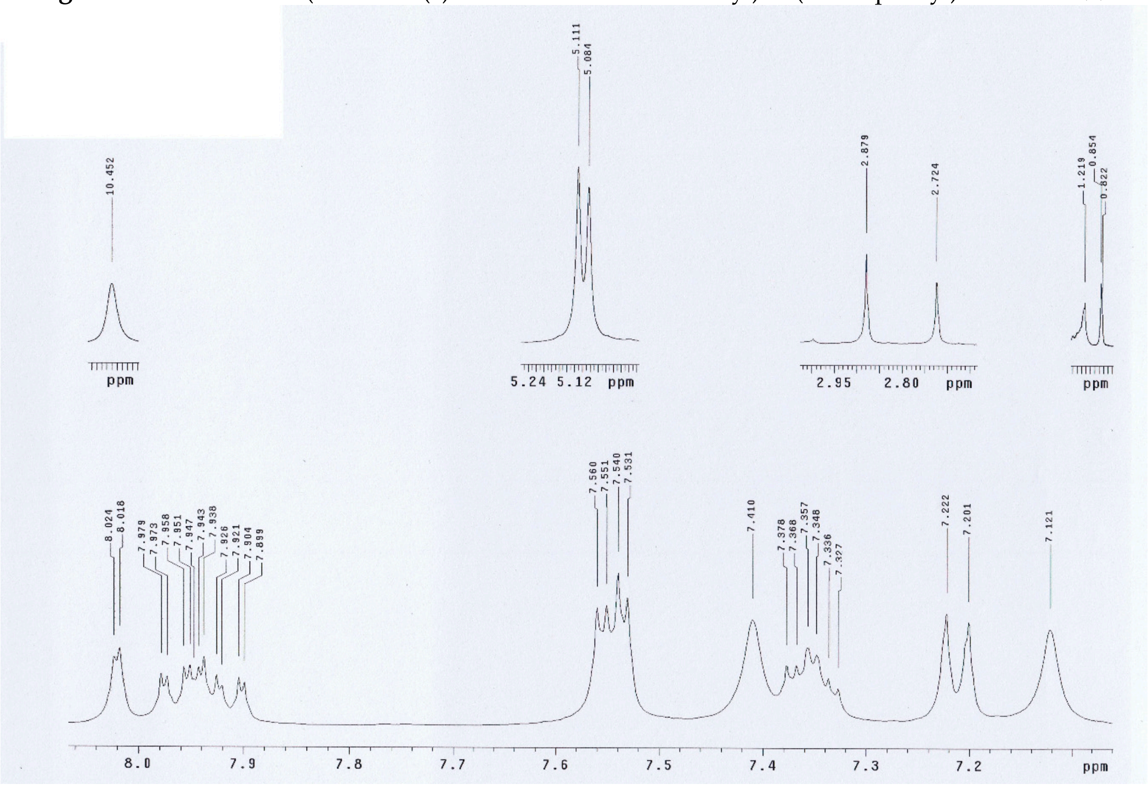

Figure S13. ^{1}H-NMR 2-(2-amino-5(6)-nitro-1*H*-benzimidazol-1-yl)-N-(2,6-dichlorophenyl) acetamide (**7**).

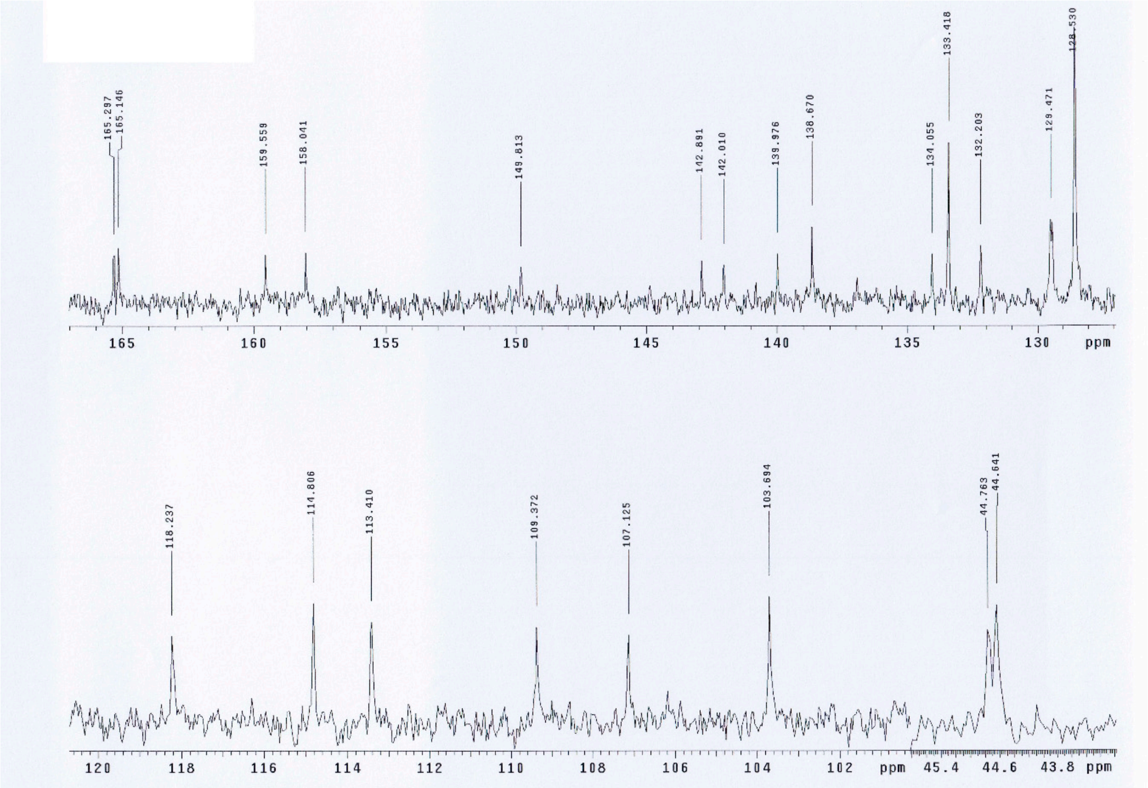

Figure S14. ^{13}C-NMR 2-(2-amino-5(6)-nitro-1*H*-benzimidazol-1-yl)-N-(2,6-dichlorophenyl) acetamide (**7**).

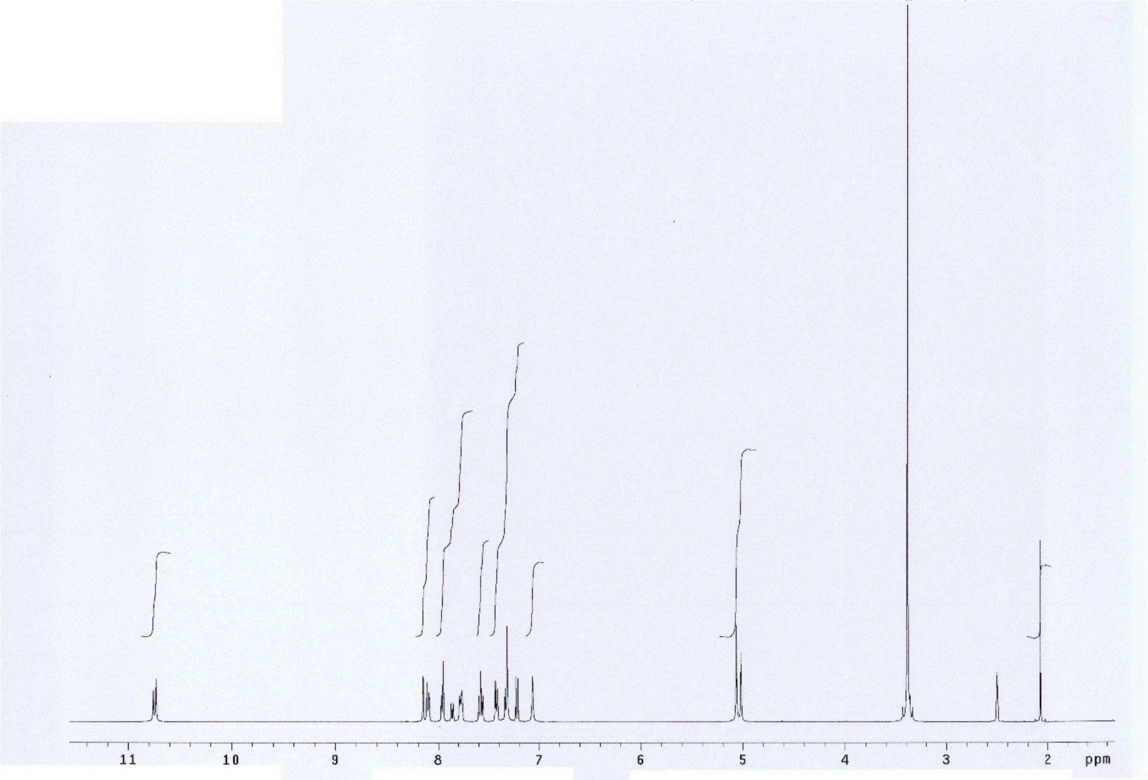

Figure S15. ^{1}H-NMR 2-(2-amino-5(6)-nitro-2,3-dihydro-1*H*-benzimidazol-1-yl)-*N*-[3-(trifluoromethyl)-phenyl]acetamide (**8**).

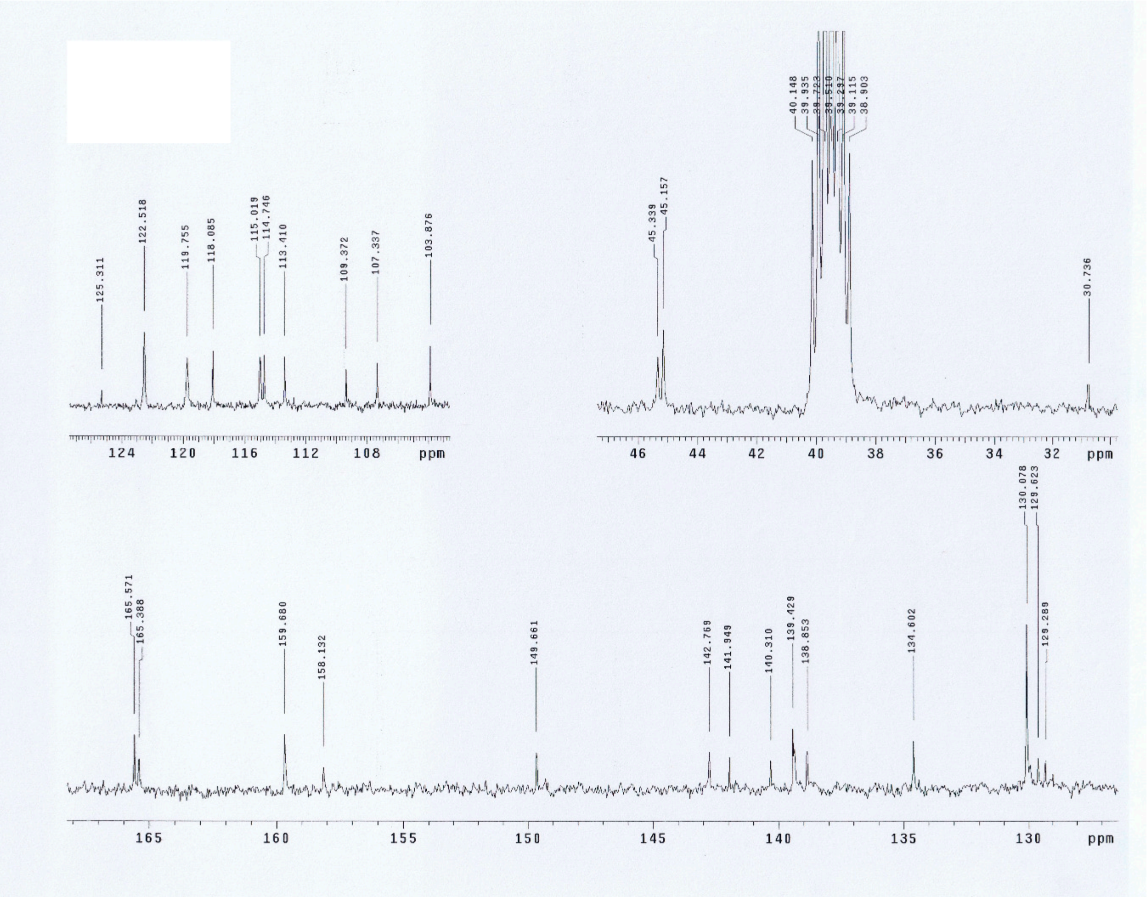

Figure S16. ^{13}C-NMR 2-(2-amino-5(6)-nitro-2,3-dihydro-1*H*-benzimidazol-1-yl)-*N*-[3-(trifluoromethyl)phenyl]acetamide (**8**).
